# Stereocontrolled synthesis of 5-azaspiro[2.3]hexane derivatives as conformationally “frozen” analogues of L-glutamic acid

**DOI:** 10.3762/bjoc.10.110

**Published:** 2014-05-14

**Authors:** Beatrice Bechi, David Amantini, Cristina Tintori, Maurizio Botta, Romano di Fabio

**Affiliations:** 1 Università degli Studi di Siena, Dipartimento Farmaco Chimico Tecnologico, Via A. Moro 2, 53100, Siena, Italy; 2 Present address: Manchester Institute of Biotechnology, School of Chemistry, University of Manchester, Oxford Road, Manchester, M13 9PL, UK; 3 Neurosciences Centre of Excellence for Drug Discovery, GlaxoSmithKline Medicines Research Centre, Via A. Fleming 4, 37135, Verona, Italy; 4 Present address: Galapagos SASU, 102 avenue Gaston Roussel, 93230 Romainville, France; 5 Present address: Drug Design and Discovery, Aptuit S.r.l., Via A. Fleming 4, 37135 Verona, Italy

**Keywords:** Amino acids, carbenes, cyclopropanation, rhodium, spiro compounds

## Abstract

Several strategies aimed to “freeze” natural amino acids into more constrained analogues have been developed with the aim of enhancing in vitro potency/selectivity and, more in general, drugability properties. The case of L-glutamic acid (L-Glu, **1**) is of particular importance since it is the primary excitatory neurotransmitter in the mammalian central nervous system (CNS) and plays a critical role in a wide range of disorders like schizophrenia, depression, neurodegenerative diseases such as Parkinson’s and Alzheimer’s and in the identification of new potent and selective ligands of ionotropic and metabotropic glutamate receptors (GluRs). To this aim, bicycle compound **Ib** was designed and synthesised from D-serine as novel [2.3]-spiro analogue of L-Glu. This frozen amino acid derivative was designed to further limit the rotation around the C3–C4 bond present in the azetidine derivative **Ia** by incorporating an appropriate spiro moiety. The cyclopropyl moiety was introduced by a diastereoselective rhodium catalyzed cyclopropanation reaction.

## Introduction

**Figure 1:**
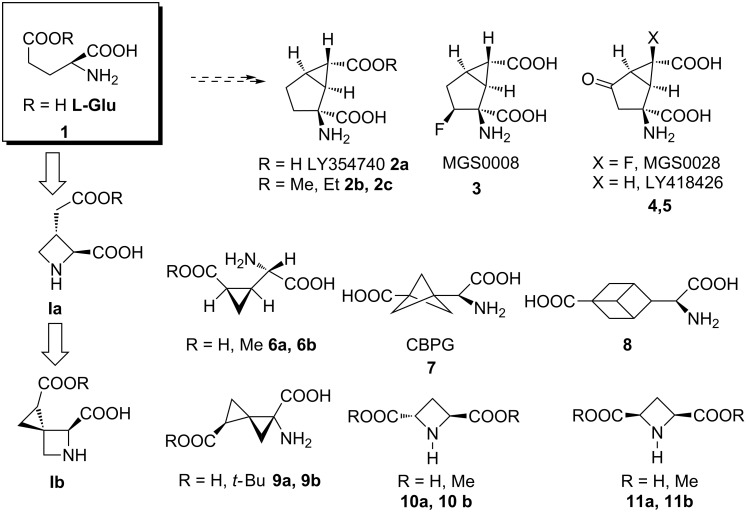
Glutamate receptor ligands.

L-Glutamic acid (L-Glu) is the primary excitatory neurotransmitter in the mammalian central nervous system (CNS) playing a critical role in the learning and memory process [1–3]. L-Glu receptors can be subdivided in ionotropic receptors (NMDA, AMPA and kainite receptors) [4–5] and G-protein coupled or metabotropic glutamate receptors (mGluRs) [6–7]. To date, eight different metabotropic receptor subtypes (mGluR1–8) have been identified. Compounds that modulate the function of the mGluRs might be useful for treating a wide range of CNS disorders including schizophrenia, depression, anxiety, addiction, pain, epilepsy and neurodegenerative diseases such as Parkinson’s and Alzheimer’s. Therefore, the identification of potent and selective mGluRs agonists and/or antagonists is critical to elucidate the role of the individual GluRs in the pathophysiology of these CNS diseases. In the last decade several potent in vitro and in vivo mGluR agonists have been reported (Figure 1).

Eglumegad (LY354740, **2a**) [8–10] was identified by Eli Lilly and investigated as a potential treatment for anxiety and drug addiction. Modifications to this molecule resulted in the identification of the analogues MGS0008 (**3**) [11] and MGS0028 (**4**) [12–13]. In addition, the conformationally constrained analogues of L-Glu **6a,b**, **7**, **8** and **9a,b** were reported [14–18] as either ionotropic or metabotropic glutamate receptors ligands, obtained by “freezing” the glutamate skeleton in search for subtype selective bioactive conformations [19]. Following the latter approach, **Ib**, shown in Figure 1, was designed as a novel potential ligand of the L-Glu receptors and building block for peptidomimetics. To the best of our knowledge, few structurally related azetidine derivatives **10a,b**,**11a,b** and **Ia** [20–22], have been reported to date. The preparation of compound **Ib** appears challenging due to both the need to control the stereochemistry of three contiguous chiral centers and the presence of a [2.3]-spiro junction connecting the cyclopropane moiety with a highly functionalized azetidine ring. Here, we describe the original synthetic approach of compound **Ib** along with the stereochemical elucidation of the diastereoisomers obtained.

## Results and Discussion

**Scheme 1:**
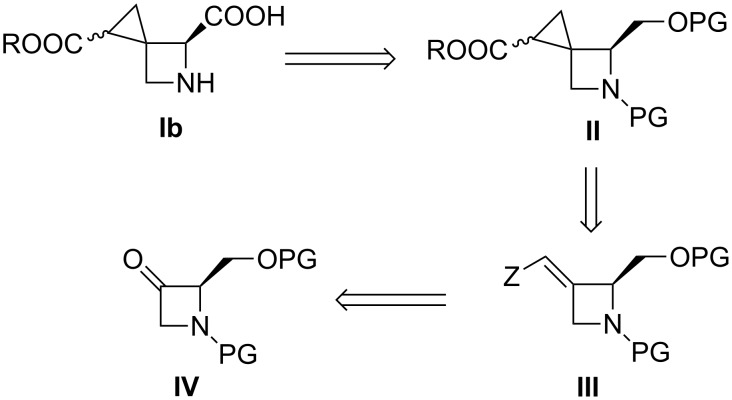
Proposed synthetic plan for the preparation of compound of type **Ib**.

It was envisioned that the synthesis of compound **Ib** could be accomplished as highlighted in Scheme 1 starting from the known ketone derivative **IV** [23–24], pursuing two different synthetic strategies: a) cyclopropanation of an α,β-unsaturated ester (compound **III**, Z = COOR); b) metal-catalyzed cyclopropanation of the corresponding terminal olefin derivative (compound **III**, Z = H) with a diazoacetate derivative.

**Scheme 2:**
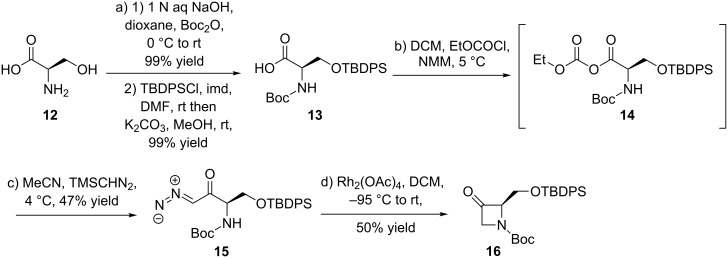
Synthesis of 3-azetidinone derivative **16**.

After having accomplished this key step, intermediate **II** would be transformed into the target compound **Ib** by sequential deprotection and oxidation of the primary alcohol to access the targeted bridged amino acid derivative. Scheme 2 shows that the synthesis started from the known azetidinone derivative **16** [23–24], whose preparation was further optimized by replacing step c) CH_2_N_2_ (diazomethane) with TMSCHN_2_ (trimethylsilyl diazomethane).

**Scheme 3:**
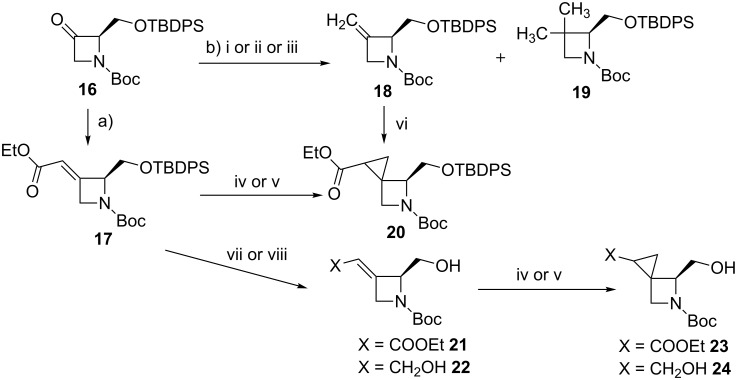
Synthetic routes to prepare target cyclopropyl derivatives **20**. Reagents and conditions: a) (EtO)_2_POCH_2_COOEt, NaH, THF, 0 °C, −78 °C, rt, 2 h 30 min, 68%; b) i. methyltriphenylphosphonium bromide, *n*-BuLi, THF, −78 °C to rt, 2 h 30 min, 23%; ii. Tebbe reagent 0.5 M in toluene, Pyr, THF, −40 °C to rt, 36%; iii. Petasis reagent, toluene, 70–90 °C in the dark, 2 h, 58%; iv. trimethylsilylsulfoxonium iodide, DBU, MeCN, 60 °C, 6 h; v. Et_2_Zn, CH_2_I_2_, DCM, 0 °C to rt, 5 days; vi. ethyl diazoacetate, Rh_2_(OAc)_4_ 10 mol %, DCM, 40 °C, 48 h, 60%*.* vii. TEA^.^3HF, TEA, THF, 50 °C, 24 h, 92%; viii. TEA^.^3HF, TEA, THF, 50 °C, 24 h, 92% then DIBAL, DCM, −78 °C to rt, 12 h, 27%.

This intermediate was transformed into intermediate **17** by a Horner–Wadsworth–Emmons reaction [23–24], thereby obtaining **17** as the single *E-*isomer in 68% yield after purification by flash chromatography (Scheme 3).

**Figure 2:**
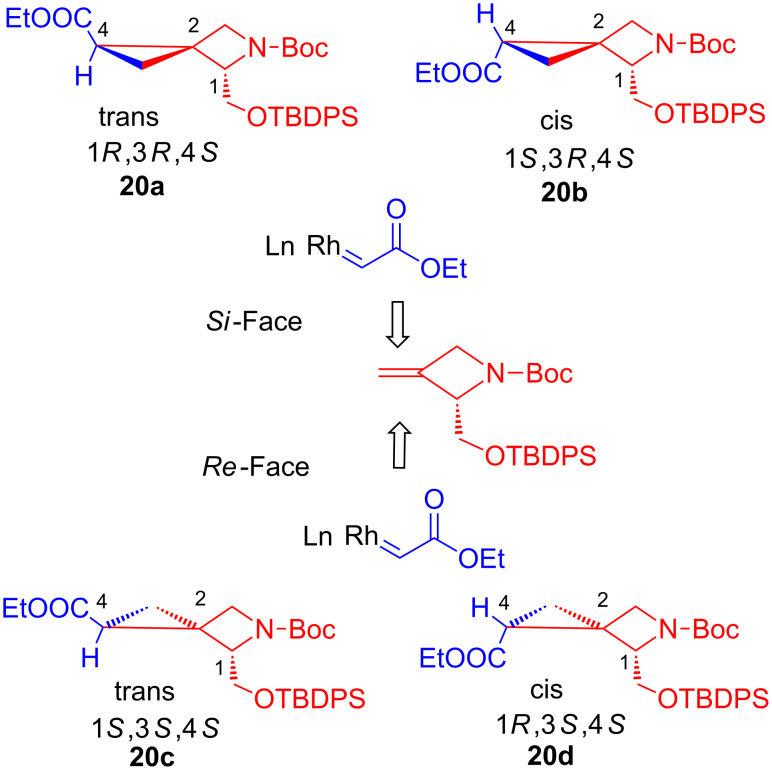
Mechanism for the attack of the carbene intermediate to the olefin moiety **18**.

Then, a systematic study of the reactivity of compound **17** was undertaken to identify the most efficient method to introduce the cyclopropane ring on the sterically hindered, α,β-unsaturated trisubstituted olefin group. With this goal in mind, both the Corey–Chaykovsky [25–27] and the Simmons–Smith [28–33] cyclopropanation reaction were attempted (highlighted in Scheme 3). Regrettably, when these reactions were performed under different reaction conditions by changing the base, the solvent, the temperature and the reaction time, only trace amounts of final product **20** were obtained. Following these initial negative results, compound **17** was de-silylated to remove the steric bulk of the protecting group and improve the reactivity towards the cyclopropanation reactions, affording compound **21**. In addition, the ester moiety was reduced with DIBAL (diisobutylaluminium hydride) to yield compound **22**. Corey–Chaykovsky cyclopropanation and Simmons–Smith cyclopropanation protocols were then performed on both derivatives **21** and **22** obtaining only trace amounts of products **23** and **24**. Based on this initial set of results, we decided to abandon the synthetic strategy a) and to explore the synthetic feasibility of approach b), namely the cyclopropanation of the corresponding terminal olefin derivative **18**. To explore this alternative approach, we managed to prepare the ethylidene derivative **18** by using either the Wittig or the Tebbe olefination reaction [34–37]. The former reaction, when accomplished in the presence of methyltriphenylphosphonium bromide and BuLi (butyllithium), successfully afforded the olefin derivative **18**, albeit in low yield (23%). The Tebbe reaction was found to be more capricious, and it worked successfully only in small scale (50 mg of compound **16**) and in the presence of a large excess of Tebbe reagent (from 4 to 8 equivalents), giving the target compound **18**, but only in limited yield (37%). However, when this reaction was scaled-up (400 mg of compound **16)**, no conversion to the desired olefin derivative **18** was observed, and, regrettably, only the byproduct **19** was isolated from the reaction mixture. To overcome this synthetic hurdle and to obtain amounts of the key intermediate **18** which are large enough to investigate its reactivity in the following cyclopropanation reaction, we decided to attempt the Petasis olefination reaction [38–39]. The initial attempts afforded compound **18** in 50% average yield, but also resulted in significant amounts of the undesired byproduct **19** (ratio **18**:**19** = 4:1 by ^1^H NMR), a compound difficult to separate by flash chromatography from product **18**. Therefore, a thorough optimization of the reaction conditions was undertaken to maximize the yield, avoiding the formation of the byproduct **19**. In particular, when the reaction was performed with a large amount of compound **16** (1.5 g) under dilute reaction conditions (0.034 M solution in toluene) by adding 3 equivalents of the Petasis reagent and stirring the reaction mixture at 70–90 °C for 2 h, the olefin derivative **18** was isolated in 58% yield after purification by flash chromatography. Notably, under these reaction conditions no formation of the byproduct **19** was observed. After successfully obtaining terminal olefin **18**, the efforts were then focused on the exploration of the reactivity of the terminal olefin towards the key cyclopropanation step performed in the presence of ethyl diazoacetate and Rh_2_(OAc)_4_ (rhodium acetate dimer). The reaction was carefully studied in different solvents (i.e., CH_2_Cl_2_, DCE, toluene) and with variable amounts of both ethyl diazoacetate and Rh_2_(OAc)_4_. In particular, encouraging results were obtained when the reaction was performed in CH_2_Cl_2_ in the presence of 1 equivalent of ethyl diazoacetate added to the reaction mixture by a syringe pump over 10 h, heated under reflux, and in the presence of a catalytic amount of Rh_2_(OAc)_4_. Under these conditions target compound **20** was obtained in poor yield (12%) as a mixture of diastereoisomers inseparable by flash chromatography. Then, the use of an excess of ethyl diazoacetate (8 equivalents) led to an increased reaction yield of up to 51%. Finally, an optimization study on both the reaction concentration (0.025 M) and the catalyst loading (10% Rh_2_(OAc)_4_), enabled us to improve the yield up to 60%. As already anticipated, compound **20** was obtained as a mixture of diasteroisomers. The HPLC analysis of the mixture revealed the presence of six diasteroisomers: two of them major (relative ratio: 49%, 33%), the others minor (12%, 3%, 1.8% and 1.2%, respectively). The presence of two unexpected additional diastereoisomers can be explained based upon a partial racemisation of the chiral center next to the nitrogen, most likely occurring during the Petasis olefination reaction of intermediate **16**. The two most abundant diastereoisomers were isolated in pure form by semi-preparative chiral HPLC and their stereochemistry was elucidated by NOE studies [40]. In principle, as shown in Figure 2, the attack of the carbene intermediate to the olefin moiety **18** can occur at both the *re* and *si* faces of the terminal olefin group, therefore affording both the *trans* and the *cis* pair of diasteroisomers.

**Figure 3:**
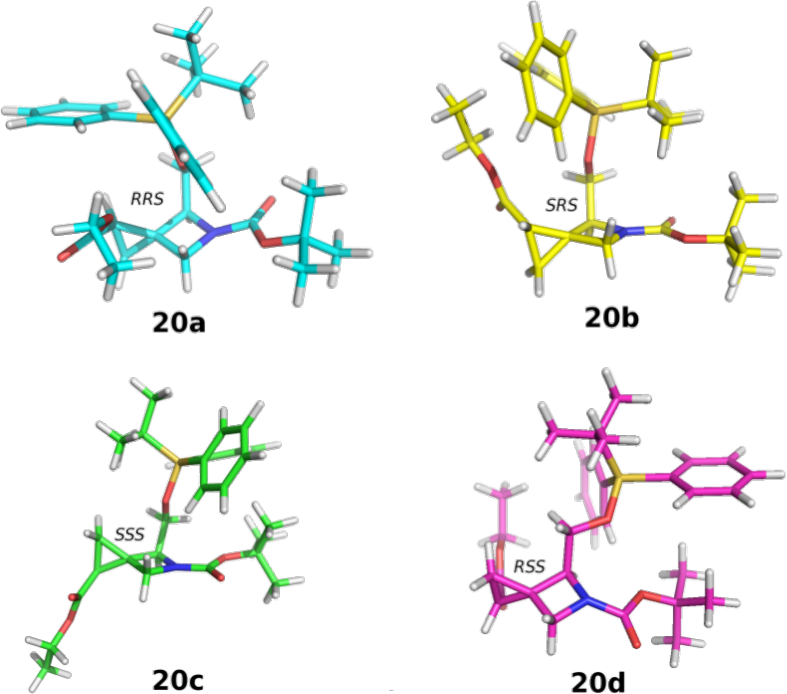
Representation of the lowest energy conformation of each diastereoisomers.

As expected based on data available in literature [41–42], the reaction was highly diastereoselective toward the formation of the two *trans* cyclopropane derivatives **20a** and **20c**. Furthermore, a partial facial selectivity was observed in favor of the S*i* face attack (ratio **20a**:**20c** = 1.5:1). To explain the results, the relative stability of the four diastereoisomers **20a–d** was assessed by theoretical calculations. 10000 conformations were generated for each molecule by using the mixed torsional/low-mode conformational sampling method in MacroModel version 9.111. The resulting geometries were minimized with the Polak–Ribiere Conjugate Gradient algorithm with OPLS-2005 as a force field until convergence to a gradient of 0.05 kJ/mol. Redundant conformers were eliminated based on a rmsd cutoff of 0.5 Å, while an energy cutoff of 5 kcal/mol was applied to discard unreasonable conformations. Default values were used for all the remaining parameters. The lowest energy conformation of the four diastereoisomers **20a–d** (Figure 3) was saved to perform the following quantum-mechanical calculations, which were obtained in vacuo at the Hartree–Fock SCF level by using a 6-31G* basis set. Finally, a full geometry optimization was carried out for each diastereoisomer by means of the Gaussian09 program [43].

The relative energies of the four diastereoisomers depicted in Figure 3 are reported in Table 1.

**Table 1:** Relative energy values (kcal/mol) of the four diastereoisomer **20a–d** calculated by the HF/631G* method.

Diasteroisomer	Relative energy value (kcal/mol)

**20a** (*RRS*)	0
**20b** (*SRS*)	1.49
**20c** (*SSS*)	0
**20d** (*RSS*)	4.48

**Scheme 4:**
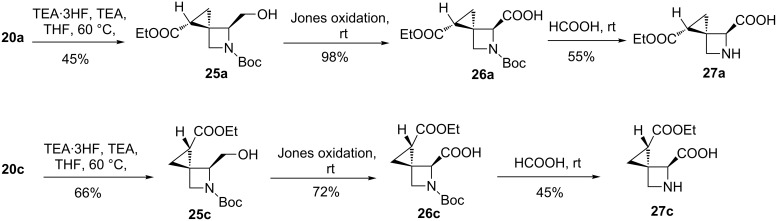
Synthesis of glutamate “frozen” analogues 4-carboxy-1-(ethoxycarbonyl)-5-azaspiro[2.3]hexane.

Compounds **20a** and **20c** showed the same level of stability and were found to be more stable than **20b** (+1.49 kcal/mol) and **20d** (+4.48 kcal/mol). These results were in line with the level of both diastereoselection and facial selectivity measured by HPLC analysis, confirming that the reaction occurred with *trans* selectivity leading to the formation of the most stable diastereoisomers. Finally, the most abundant compounds **20a** and **20c** were deprotected by triethylamine trihydrofluoride and triethylamine in THF at 60 °C, to give compounds **25a** and **25c**, which were oxidized with Jones reagent, to afford acids **26a** and **26c**. The final cleavage of the Boc protecting group was carried out in the presence of formic acid at room temperature, affording the target amino acid derivatives **27a** and **27c** (Scheme 4).

## Conclusion

In conclusion, two complex bridged analogues **27a,c** of glutamic acid were synthesized. Starting from D-serine, their synthesis was accomplished in 10 steps in good overall yield. After an extensive investigation on the best synthetic approach, key intermediate **20** was successfully prepared by an efficient rhodium-catalyzed cyclopropanation of a terminal double bond of compound **18** with ethyl acetate. The cyclopropanation reaction occurred with *trans* selectivity preferentially affording the two *trans* cyclopropane products. Theoretical calculations on the stability of the four possible diastereoisomers were in agreement with both literature and experimental data observed. The final constrained amino acid derivatives **27a** and **27c** represent useful unnatural amino acid derivatives for both peptidomimetic synthesis and as ligands of the plethora of glutamate receptors.

## Supporting Information

Experimental section comprising the synthesis of all newly synthesized compounds and intermediates, NOE studies and HPLC analysis on compounds **20a** and **20c**, Gaussian input files for QM calculations for compounds **20a**, **20b**, **20c** and **20d**, and copies of ^1^H and ^13^C NMR spectra for all new compounds.

File 1 Experimental section.

File 2 NOe studies and HPLC analysis on compounds **20a** and **20c**.

File 3 Gaussian input files for QM calculations for compounds **20a**, **20b**, **20c** and **20d**.

File 4 Copies of ^1^H and ^13^C NMR spectra for all new compounds.
