# Supplementary material for: Stereocontrolled synthesis of 5-azaspiro[2.3]hexane derivatives as conformationally “frozen” analogues of L-glutamic acid
Source: Beilstein J Org Chem. 2014 May 14;10:1114–20. doi: 10.3762/bjoc.10.110 (PMC4077356; doi:10.3762/bjoc.10.110)
Supplement: File 1 — Experimental section. [file Beilstein_J_Org_Chem-10-1114-s001.pdf]

Supporting Information File 1

for

Stereocontrolled synthesis of 5-azaspiro[2.3]hexane derivatives as conformationally “frozen” analogues of L-glutamic acid

Beatrice Bechi[1,2], David Amantini[3,4], Cristina Tintori[1], Maurizio Botta*[1] and Romano di Fabio*[3,5]

Address: [1]Università degli Studi di Siena, Dipartimento Farmaco Chimico Tecnologico, Via A. Moro 2, 53100, Siena, Italy; [2]Present address: Manchester Institute of Biotechnology, School of Chemistry, University of Manchester, Oxford Road, Manchester, M13 9PL, UK, [3]Neurosciences Centre of Excellence for Drug Discovery, GlaxoSmithKline Medicines Research Centre, Via A. Fleming 4, 37135, Verona, Italy, [4]Present address: Galapagos SASU, 102 avenue Gaston Roussel, 93230 Romainville, France and [5]Present address: Drug Design and Discovery, Aptuit S.r.l., Via A. Fleming 4, 37135 Verona, Italy

Email: Maurizio Botta - botta.maurizio@gmail.com, Romano di Fabio - romano.difabio@aptuit.com

* Corresponding author

Experimental section

Reagents were obtained from commercial suppliers and used without further purifications. The Petasis reagent was synthesized according to known procedure [1,2]. According to standard procedures, CH_2Cl_2 and CH_3CN were dried over calcium hydride, THF and toluene were dried over Na/benzophenone prior to use, and anhydrous DMF was purchased directly. Anhydrous reactions were run under a positive pressure of dry N_2 or argon. TLC was carried out using TLC plates Kieselgel 60 F_{254}. Chromatographic purifications were performed on columns packed with 60 silica gel, 23–400 mesh, for flash technique. Reactions with slow addition of reagents were performed using a syringe pump. The −95 °C temperature was approximate as achieved by a liquid nitrogen–methanol bath. Melting points were taken using a melting point apparatus. ^{1}H NMR and ^{13}C NMR spectra were measured at 200 MHz, 400 MHz and at 600 MHz spectrometers; chemical shifts are reported in δ values, relative to TMS at δ 0.00 ppm. CD_3OD, DMSO-d_6, D_2O and CD_3Cl were used as solvents. Optical rotations were measured on a polarimeter equipped with a sodium lamp (λ = 589 nm) and a 10 cm microcell. Mass spectral (MS) data were obtained using a LC/MSD VL system with a 0.4 mL/min flow rate using a binary solvent system of 95:5 MeOH/water and an electrospray ionization source (ESI). UV detection was monitored at 254 nm. All solvents were HPLC grade. Mass spectra were acquired in positive and negative mode scanning over the mass range of 50–1500. The following ion source parameters were used: drying gas flow, 9 mL min^{-1}; nebulizer pressure, 40 psi; drying gas temperature, 350 °C. Accurate mass (HRMS) data were obtained using a thermofisher system (resolution of 30000). The diastereomeric ratio for compound **20** was assessed by reversed-phase liquid chromatography LC/MSD VL system using a (*S,S*)-Whelk- O1 chiral column with a mobile phase composed of methanol/water (75:25) and a flow rate of 1.5 mL/min.

The diastereoisomeric mixture **20** was resolved on a HPLC system equipped with an adsorbance detector dual l using a semi-preparative chiral column (Regis, (*R,R*)-Whelk-O 1 5/100 25 cm × 10 mm), with a mobile phase composed of MeOH/H_2O, 70:30 at a flow rate of 10 mL/min. The sample was dissolved in a mixture MeOH/H_2O, 80:20. Diastereoisomic purity of **20a** and **20c** was established by reinjecting every single compound in the (*S,S*)-Whelk-O 1 chiral column with a mobile phase composed of methanol/water (75:25) and a flow rate of 1.5 mL/min (HPLC system).

Procedures

(3*R*)-1-Diazo-3-(*N*-*tert*-butyloxycarbonyl)amino-4-*tert*-butyldiphenylsilyloxy-2-butanone (15): To a solution of *N*-Boc-*O*-TBDPS-(*R)*-serine **13** (9.50 g, 21.415 mmol) in dichloromethane (215 mL) at −5 °C was added *N*-methylmorpholine (2.058 g, 20.350 mmol) and then ethyl chloroformate (2.557 g, 23.560 mmol). After stirring for 1 h at −5 °C the reaction was washed with cold diluted citric acid (10%), aqueous $NaHCO_3$, water, then dried (Na_2SO_4) and concentrated under reduced pressure to give a pale-yellow oil. Acetonitrile (60 mL) was added to the oil, followed by 21.4 mL (42.830 mmol) of trimethylsilyldiazomethane (2.0 M solution in hexane, Aldrich). The mixture was stirred for 43 h at 4 °C. Afterwards diethyl ether was added and the mixture was extracted with 10% aqueous citric acid (2 times), saturated aqueous $NaHCO_3$ (2 times) and brine. The organic phase was dried over Na_2SO_4, filtered and evaporated under vacuum [3-6]. The residue was subjected to column chromatography (Et_2O:hexane 2:8), to afford the 4.64 g of desired product (yellow solid, 47% yield).

^{1}H NMR, ^{13}C NMR and mass analysis for compound **15** are identical to those reported in the literature [5,6].

(2*S*,*E*)-1-*tert*-Butyloxycarbonyl-2-*tert*-butyldiphenylsilyloxymethyl-3-(2-ethoxy-2-oxoethylidene)azetidine (17): Sodium hydride (0.079 g, 3.28 mmol) was suspended in THF (10 mL) and the suspension was cooled to 0 °C under an argon atmosphere. Triethylphosphonoacetate (0.830 g, 3.70 mmol) was added to the stirred sodium hydride/tetrahydrofuran suspension over a period of 5–10 min. The mixture was cooled to −78 °C and a solution of (2*R*)-1-*tert*-butyloxycarbonyl-2-*tert*-butyldiphenylsilyloxymethylene-3-azetidinone (**16**, 0.930 g, 2.115 mmol) in THF (40 mL) was added over a period of 5–10 min. After that the mixture was stirred for 15 min at −78 °C, it was allowed to warm to room temperature and stirred for another 2 h 30 min. The reaction was quenched with saturated ammonium chloride solution and transferred to a separatory funnel with ether. The aqueous phase was separated and extracted with ether (3 times). The combined organic layers were washed with a mixture of saturated sodium hydrogen carbonate/brine (1:1), dried over sodium sulfate, filtered, and evaporated to dryness to give a yellowish oil. The crude product was purified by column chromatography over silica with dichloromethane as eluent to yield the product as a yellow oil (0.735 g, yield: 68%). The product is reported in the literature as *E*/*Z* mixture [5,6].

$[\alpha]^{25}_{D}$ = - 12.8 (c=0.94, $CHCl_3$). ^{1}H NMR (400 MHz, $CDCl_3$, 25°C): *δ*: 1.06 (s, 9H), 1.30 (t, *J* = 7.1 Hz, 3H) 1.42 (s, 9H), 3.87 (d, *J* = 9.9 Hz, 1H) 4.02-4.12 (br s, 1H), 4.21 (q, *J* = 7.0 Hz, 2H), 4.78-4.83 (m, 3H), 5.86 (s, 1H), 7.39-7.43 (m, 6H), 7.64-7.69 (m, 4H). ^{13}C NMR (100 MHz, $CDCl_3$, 25°C): *δ*:14.4, 19.3, 26.7, 28.3, 58.5, 60.3, 63.1, 70.5, 79.9, 113.6, 127.6, 129.7, 129.8, 133.1, 135.6, 155.5, 165.3 ppm. MS-

ESI (*m/z*) 532.0 $[M+Na]^+$. HRMS-ESI (*m/z*) calcd for $C_{29}H_{40}NO_5Si$ $[M + H]^+$ 510.2670, found 510.2671 (Δ=0.06 ppm).

(2*S*)-1-*tert*-Butyloxycarbonyl-2-*tert*-butyldiphenylsilyloxymethyl-3-methylideneazetidine (18): Method A [7-10]: 0.040 g (0.091mmol) of (2*R*)-1-*tert*-butyloxycarbonyl-2-*tert*-butyldiphenylsilyloxymethylene-3-azetidinone (**16**) was dissolved in 4 mL of THF and 26 µL of pyridine was added. The reaction was cooled to −40 °C. After that, 0.728 mL (0.364 mmol) of a 0.5 M solution of Tebbe reagent in toluene was added dropwise over 1–3 min and the reaction was maintained at −40 °C for 0.5 h and then allowed to warm to room temperature over an additional 60 min period. The reaction was quenched by the dropwise addition of 0.5 mL of 15% aqueous sodium hydroxide solution to the cooled (ca. −10 °C) reaction mixture. After warming to room temperature the resulting red solution was stirred at rt for 12 h to allow the complete decomposition of the aluminium and titanum salts. The resulting yellow suspension was filtered through a celite pad and washed with Et_2O. Upon solvent removal in vacuo the crude alkene was purified by silica gel column chromatography employing hexane/Et_2O 8:2 as an eluant, to obtain 0.014 g of the pure product as a yellow oil (36% yield). Scale-up of the reaction (to 0.93 mmol scale) led to the formation of the side-product **19**.

Method B [11,12]: A solution of dimethyltitanocene (3.6% w/w solution in toluene, 10 mmol, 65 mL) was added to a solution of (2*R*)-1-*tert*-butyloxycarbonyl-2-*tert*-butyldiphenylsilyloxymethylene-3-azetidinone (**16**, 1.470 g, 3.34mmol) in 100 mL of toluene. The red/orange mixture was heated to 80 °C and was aged in the dark under argon atmosphere for 2 h, then cooled to ambient temperature. Sodium hydrogen carbonate (5.2 mmol), methanol (7.0 mL) and water (0.27 mL) were added, and the mixture was heated to 40 °C for 12 h. (The hot aqueous methanol

treatment was done to decompose the titanium residues into an insoluble solid. The decomposition was judged to be complete when gas evolution ceased.) The green-yellow mixture was cooled to ambient temperature and the titanium residues were removed by filtration. The solution was evaporated under reduced pressure and the residue purified by flash chromatography on silica gel (Et_2O:hexane 15:85), affording the product as a pale yellow oil (0.850 g, 58% yield).

$[\alpha]^{25}_D$ = - 3.3 (c=1.2, $CHCl_3$). ^{1}H NMR (400 MHz, $CDCl_3$): *δ:* 1.09 (s, 9H), 1.43 (s, 9H), 3.86-3.89 (dd, *J* = 10.3, 2.8 Hz, 1H) 4.98-4.08 (br s, 1H), 4.43-4.50 (m, 2H), 4.76 (s, 1H), 5.08 (s, 1H), 5.15 (s, 1H),7.37-7.45 (m, 6H), 7.69-7.73 (m, 4H). ^{13}C NMR (100 MHz, $CDCl_3$): *δ:* 19.2, 26.7, 28.3, 56.5, 63.5, 70.6, 79.4, 106.9, 127.4, 129.6, 133.4, 135.6, 141.0, 155.9 ppm. MS-ESI (*m/z*) 460 $[M+Na]^+$, 476 $[M+K]^+$. HRMS-ESI (*m/z*) calcd for $C_{26}H_{36}NO_3Si$ $[M + H]^+$ 438.2459, found 438.2463 (Δ=0.94 ppm).

(2*S*)-1-*tert*-Butyloxycarbonyl-2-*tert*-butyldiphenylsilyloxymethyl-3,3'-dimethyl-azetidine (19): (2*S*)-1-*tert*-Butyloxycarbonyl-2-*tert*-butyldiphenylsilyloxymethyl-3,3'-dimethylazetidine (**19**) was isolated as the single product when preparing (2*S*)-1-*tert*-butyloxycarbonyl-2-*tert*-butyldiphenylsilyloxymethyl-3-methylideneazetidine (**18**) with **method A** in 0.93 mmol scale (0.408 g). 0.232 g (55% yield) of a colorless oil were recovered. $[\alpha]^{25}_D$ = - 8.8 (c=1.6, $CHCl_3$). ^{1}H NMR (400 MHz, $CDCl_3$, 25°C): *δ:* 1.12 (s, 9H), 1.35 (s, 6H), 1.36 (s, 9H), 3.48-3.57 (dd, *J* = 28.9, 7.8 Hz, 2H) 3.90-3.94 (m, 2H), 4.00-4.04 (m, 1H), 7.36-7.44 (m, 6H), 7.71-7.74 (m, 4H). ^{13}C NMR (100 MHz, $CDCl_3$, 25°C): *δ:* 19.2, 21.8, 26.9, 28.3, 28.8, 33.7, 59.8, 62.3, 70.0, 79.2, 127.7, 129.7, 134.9, 135.5, 156.8 ppm. MS (ESI): *m/z* 476.2 $[M+Na]^+$.

(4*S*)-5-*tert*-Butyloxycarbonyl-4-(*tert*-butyldiphenylsilyloxymethyl)-1-ethyloxycarbonyl-5-azaspiro[2.3]hexane (20): A 0.025 M solution of (2*S*)-1-*tert*-

butyloxycarbonyl-2-*tert*-butyldiphenylsilyloxymethyl-3-methylideneazetidine (**18**, 0.253 g, 0.578 mmol) in dry and degassed DCM (23 mL) was charged with $Rh_2(OAc)_4$ (0.026 g, 10 mol %). The reaction was stirred at reflux for 30 min. A 0.1 M solution of ethyl diazoacetate (0.527 g, 4.62 mmol) in dry and degassed DCM (46 mL) was added dropwise to the refluxing solution with a syringe pump over a period of 21 h. The reaction was stirred at reflux for 2 days [13-16]. The solution was evaporated under reduced pressure and the residue purified by flash chromatography on silica gel (Et_2O:hexane 15:85), affording the product as a pale yellow oil (0.180 g, 60%, mixture of diastereomers).

^{1}H NMR (400 MHz, $CDCl_3$, 25°C): *δ:* 0.78-1.59 (m), 3.42-4.21 (m), 7.14-7.19 (m) 7.41-7.50 (m). MS-ESI (*m/z*)546.2 $[M+Na]^+$, 562.2 $[M+K]^+$.HRMS-ESI (*m/z*) calcd for $C_{30}H_{42}NO_5Si$ $[M + H]^+$ 524.2827, found 524.2829. (Δ=0.38 ppm).

20 was obtained as mixture of 6 diastereoisomers. The diastereoisomeric mixture **20** was resolved on a Waters 2525 HPLC system equipped with a adsorbance detector dual l using a semipreparative chiral column (Regis, (*R,R*)-Whelk-O 1 5/100 25 cm × 10 mm, with a mobile phase composed of MeOH/H_2O, 70:30 at a flow rate of 10 mL/min. The sample was dissolved in a mixture MeOH:H_2O, 80:20.

(1*R*,3*R*,4*S*)-5-tert-Butyloxycarbonyl-4-(*tert*-butyldiphenylsilyloxymethyl)-1-ethyloxycarbonyl-5-azaspiro[2.3]hexane (20a): HPLC Rt 10.00 min, 49%; $[\alpha]^{20}_D$= -30 (c=1, $CHCl_3$). ^{1}H NMR (400 MHz, $CDCl_3$): δ: 1.10 (s, 9H), 1.20-1.26 (m, 4H), 1.34-1.37 (m, 1H), 1.41 (s, 9H), 1.76-1.80 (dd, *J* = 8.3, *J* = 5.8 Hz, 1H), 3.68 (d, *J* = 10.7 Hz, 1H), 3.93 (d, *J* = 7.6 Hz, 1H), 4.05-4.12 (m, 4H), 4.21 (br s, 1H), 7.36-7.43 (m,6H), 7.69-7.71 (m, 4H). ^{13}C NMR (100 MHz, $CDCl_3$): δ 14.3, 16.6, 19.3, 23.3, 26.5, 26.88, 28.4, 54.8, 60.6, 62.1, 67.2, 79.4, 127.61, 127.65, 129.57, 129.64,

133.4, 133.8, 135.6, 135.7, 155.4, 172.1. MS-ESI (*m/z*) 546.2 $[M+Na]^+$, 562.2 $[M+K]^+$. HRMS-ESI (*m/z*) calcd for $C_{30}H_{42}NO_5Si$ $[M + H]^+$ 524.2827, found 524.2829 (Δ=0.35 ppm).

(1*S*,3*S*,4*S*)-5-*tert*-Butyloxycarbonyl-4-(*tert*-butyldiphenylsilyloxymethyl)-1-ethyloxycarbonyl-5-azaspiro[2.3]hexane (20c): HPLC Rt 12.30 min, 33%; $[\alpha]^{20}_D$ = +43 (c=1, $CHCl_3$). ^{1}H NMR (400 MHz, $CDCl_3$, 25°C): δ: 1.05 (s, 9H), 1.22-1.24 (m, 1H), 1.28 (t, *J* = 7.1 Hz, 3H), 1.36 (s, 9H), 1.56 (br s, 1H), 1.77-1.81 (dd, *J* = 8.5, *J* = 5.7 Hz, 1H), 3.78 (d, *J* = 9.4 Hz, 1H), 3.90-4.00 (m, 3H), 4.16 (d, *J* = 7.1 Hz, 2H), 4.26 (d, *J* = 4.9 Hz, 1H), 7.36-7.44 (m,6H), 7.62-7.66 (m, 4H). ^{13}C NMR (100 MHz, $CDCl_3$, 25°C):δ 14.3, 15.0, 18.6, 19.1, 22.9, 26.5, 28.7, 53.9, 60.9, 63.1, 66.4, 79.6, 127.68, 127.7, 127.8, 129.7, 129.8, 133.15, 133.20, 135.47, 135.51, 135.54, 155.8, 172.0. MS-ESI (*m/z*)546.2 $[M+Na]^+$, 562.2 $[M+K]^+$. HRMS-ESI (*m/z*) calcd for $C_{30}H_{42}NO_5Si$ $[M + H]^+$ 524.2827, found 524.2829 (Δ=0.35 ppm).

(2*S*,*E*)-1-*tert*-Butyloxycarbonyl-2-hydroxymethyl-3-(2-ethoxy-2-oxoethylidene)azetidine (21): A mixture of triethlyamine trihydrofluoride (0.582 g, 3.6 mmol) and triethylamine (0.183 g, 1.8 mmol) in 5 mL of THF was added to (2*S*,*E*)-1-*tert*-butyloxycarbonyl-2-*tert*-butyldiphenylsilyloxymethyl-3-(2-ethoxy-2-oxoethylidene)azetidine (**17**, 0.263 g, 0.5 mmol) in THF (10 mL). The reaction mixture was stirred at 50 °C for 24 h. The solvent was removed under reduced pressure and the residue obtained was purified by flash silica gel column chromatography and eluted with hexane:Et_2O (1:1) to afford (2*S*,*E*)-1-*tert*-butyloxycarbonyl-2-hydroxymethyl-3-(2-ethoxy-2-oxoethylidene)azetidine (**21**) as a colourless oil (0.128 g, 92%):

$[\alpha]^{25}_{D}$ = + 3 (c=1, $CHCl_3$). ^{1}H NMR (400 MHz, $CDCl_3$, 25°C): *δ:* 1.21 (t, *J* = 7.0 Hz, 3H), 1.40 (s, 9H), 3.78-3.82 (m, 2H), 4.11 (q, *J* = 7.0 Hz, 2H), 4.64-4.73 (m, 2H), 4.90 (br s, 1H), 5.75 (s, 1H). ^{13}C NMR (100 MHz, $CDCl_3$, 25°C): *δ:* 14.2, 28.3, 29.7, 58.5, 60.5, 64.5, 71.8, 81.1, 114.0, 152.5, 165.0 ppm. MS-ESI (*m/z*) 294.0 $[M+Na]^+$. HRMS-ESI (*m/z*) calcd for $C_{13}H_{22}NO_5$ $[M + H]^+$ 272.1492, found 272.1497 (Δ=1.47 ppm).

(2*S*,*E*)-1-*tert*-Butyloxycarbonyl-2-hydroxymethyl-3-(2-hydroxy-2-ethylidene)azetidine (22): To a solution of (2*S*,*E*)-1-*tert*-butyloxycarbonyl-2-hydroxymethyl-3-(2-ethoxy-2-oxoethylidene)azetidine (**21**, 0.079 g, 0.29 mmol) in DCM (10 mL) at −78 °C under argon atmosphere, was added slowly a 1 M solution of DIBAL in DCM (2.9 mL, 2.9 mmol). The reaction mixture was stirred at −78 °C for 1 h, and at room temperature for 12 h. After that, EtOAc (10 mL) was added and the solution was stirred for 10 min to destroy the unreacted DIBAL. Dichloromethane was removed under reduced pressure; the organic phase solution was washed with a saturated solution of Rochelle salt and H_2O, dried (Na_2SO_4), and concentrated. Flash chromatography of the residue (DCM:MeOH 9:1) furnished (2*S*,*E*)-1-*tert*-butyloxycarbonyl-2-hydroxymethyl-3-(2-ethoxy-2-oxoethylidene)azetidine (**22**) as a colorless oil (0.018 g, 27%).

^{1}H NMR (400 MHz, $CDCl_3$, 25°C): *δ:* 1.45 (s, 9H), 3.70 (m,2H), 4.10 (m, 2H), 4.45 (bs, 2H), 4.85 (br s, 1H), 5.5 (bs, 1H). MS (ESI): (*m/z*)251.9 $[M+Na]^+$. HRMS-ESI (*m/z*) calcd for $C_{11}H_{20}NO_4$ $[M + H]^+$ 230.1387, found 230.1390 (Δ=1.46 ppm).

(1*R*,3*R*,4*S*)-5-*tert*-Butyl 1-ethyl 4-(hydroxymethyl)-5-azaspiro[2.3]hexane-1,5-dicarboxylate (25a): A mixture of triethlyamine trihydrofluoride (0.138 g, 0.86 mmol) and triethylamine (0.044 g, 0.43 mmol) in THF (1 mL) was added to (1*R*,3*R*,4*S*)-5-

tert-butyloxycarbonyl-4-(*tert*-butyldiphenylsilyloxymethyl)-1-ethyloxycarbonyl-5 azaspiro[2.3]hexane (**20a**, 0.045 g, 0.086 mmol) in THF (2 mL). The reaction mixture was stirred at 60 °C for 48 h. Since the reaction was not complete, triethlyamine trihydrofluoride (0.069 g, 0.43 mmol) and triethylamine (0.022 g, 0.215 mmol) were added, and the reaction was stirred at 60 °C for an additional 3 days. After that, 0.86 mmol (0.138 g) of triethlyamine trihydrofluoride and 0.43 mmol (0.044 g) of triethylamine were added, and the reaction stirred at 60 °C for additional 6 h H_2O and Et_2O were added, and the phases were separated. Organic phase was washed with H_2O (2 times), dried over Na_2SO_4 and removed under reduced pressure. The residue obtained was purified by flash silica gel column chromatography and eluted with hexane:Et_2O (4:6) to afford (1*R*,3*R*,4*S*)-5-*tert*-butyloxycarbonyl-4-(hydroxymethyl)-1-ethyloxycarbonyl-5-azaspiro[2.3]hexane (**25a**) as a colourless oil (0.011 g, 45%)

$[\alpha]^{20}_{D}$ = - 53.2 (c=1.09, $CHCl_3$). ^{1}H NMR (400 MHz, $CDCl_3$, 25°C): *δ:* 1.23-1.27 (m, 4H), 1.40-1.48 (m, 10H), 1.70-1.73 (m, 1H), 3.80-3.83 (m, 3H), 3.99 (d, *J* = 8 Hz, 1H), 4.10-4.16 (ddd, *J* =4 Hz, *J* =8 Hz, *J* =12 Hz, 2H), 4.37 (bs, 1H); ^{13}C NMR ($CDCl_3$, 100 MHz, 25°C) δ: 14.3, 15.2, 22.2, 25.6, 28.2, 54.7, 61.15, 64.3, 66.8, 80.6, 156.4, 172.0. MS (ESI) (*m/z*)308.1 $[M+Na]^+$.HRMS-ESI (*m/z*) calcd for $C_{14}H_{24}NO_5$ $[M + H]^+$ 286.1649, found 286.1654 (Δ=1.61 ppm).

(1*S*,3*S*,4*S*)-5-*tert*-Butyl 1-ethyl 4-(hydroxymethyl)-5-azaspiro[2.3]hexane-1,5-dicarboxylate (25c): Compound **25c** was synthesised using the same procedure described for compound **25a**, using as starting material (1*S*,3*S*,4*S*)-5-*tert*-butyloxycarbonyl-4-(*tert*-butyldiphenylsilyloxymethyl)-1-ethyloxycarbonyl-5-azaspiro[2.3]hexane (**20c**, 0.043 g, 0.082 mmol), adding respectively 10 equiv (0.132

g, 0.82 mmol) of TEA·HF and 5 equiv (0.041 mg, 0.41 mmol) of TEA and stirring the reaction at 60 °C for 41 h.

Colourless oil. 0.016 g, 66% yield. $[\alpha]^{20}_{D}$ = + 296.5 (c=0.58, $CHCl_3$). ^{1}H NMR (400 MHz, $CDCl_3$, 25°C): δ: 1.16-1.23 (m, 1H), 1.25 (t, *J* =6Hz, 3H), 1.43 (s, 10H), 1.69-1.73 (dd, *J* = 4Hz, *J* = 8Hz, 1H), 3.52 (bs, 1H), 3.69-3.75 (m, 1H), 3.90-4.01 (dd, *J* =12Hz, *J*=16Hz, 2H), 4.09-4.15 (ddd, *J* =4 Hz, *J* =8 Hz, *J* =12 Hz, 2H), 4.39 (bs, 1H); ^{13}C NMR ($CDCl_3$, 100 MHz, 25°C) δ: 14.3, 14.5, 21.6, 25.9, 28.3, 54.2, 60.8, 64.4, 67.2, 80.7, 138.2, 171.7. MS-ESI (*m/z*)308.1 $[M+Na]^+$. HRMS-ESI (*m/z*) calcd for $C_{14}H_{24}NO_5$ $[M + H]^+$ 286.1649, found 286.1654 (Δ=1.60 ppm).

(1*R*,3*R*,4*S*)-5-(*tert*-Butoxycarbonyl)-1-(ethoxycarbonyl)-5-azaspiro[2.3]hexane-4-carboxylic acid (26a): To a solution of (1*R*,3*R*,4*S*)-5-*tert*-butyl 1-ethyl 4-(hydroxymethyl)-5-azaspiro[2.3]hexane-1,5-dicarboxylate (**25a**, 0.011 g, 0.039 mmol) in acetone (0.38 mL) at 0 °C was added chromic acid (0.15 mL, 2.6 M solution, 0.386 mmol). The resulting reaction mixture was stirred at rt for 1 h, then 0.05 mL of 2.6 M solution of chromic acid was added and the reaction stirred for an additional hour at rt. Isopropanol was added to destroy the residual chromic acid followed by the addition of water. The mixture was washed with H_2O (3 times) and the organic phase was dried (Na_2SO_4), filtered, and the filtrate was concentrated in vacuum to dryness. Compound (1*R*,3*R*,4*S*)-5-*tert*-butyloxycarbonyl-4-carboxylic acid-1-ethyloxycarbonyl-5-azaspiro[2.3]hexane (**26a**, colourless oil) was submitted to the next step of the synthesis without further purifications. $[\alpha]^{20}_{D}$ = -42.3 (c=1.3, $CHCl_3$). ^{1}H NMR (400 MHz, $CDCl_3$, 25°C): *δ:* 0.8-0.89 (m, 1H), 1.29-1-32 (m, 4H), 1.43 (s, 9H), 1.92-1.97 (m, 1H), 4.01 (s, 2H), 4.20-4.24 (q, *J* =4 Hz, 2H), 4.75 (bs, 1H); ^{13}C NMR ($CDCl_3$, 100 MHz, 25°C) δ: 14.1, 16.6, 22.6, 27.6, 28.2, 54.1, 62.4, 64.3, 81.4, 155.7, 169.0,

174.2. MS-ESI (*m/z*)322.0 $[M+Na]^+$, 298.2 $[M-H]^-$. HRMS-ESI (*m/z*) calcd for $C_{14}H_{22}NO_6$ $[M + H]^+$ 300.1442, found 300.1447 (Δ=1.62 ppm).

(1*S*,3*S*,4*S*)-5-(*tert*-Butoxycarbonyl)-1-(ethoxycarbonyl)-5-azaspiro[2.3]hexane-4-carboxylic acid (26c): Compound **26c** was synthesized following the same procedure described for compound **26a**, using as starting material compound (1*S*,3*S*,4*S*)-5-*tert*-butyl 1-ethyl 4-(hydroxymethyl)-5-azaspiro[2.3]hexane-1,5-dicarboxylate (**25c**, 0.016 g, 0.056 mmol). Compound **26c** (colourless oil) was submitted to the next step of the synthesis without further purifications. $[\alpha]^{20}_D$ = +22.5 (c=1.2, $CHCl_3$). ^1H NMR (400 MHz, $CDCl_3$): *δ:* 0.81-0.90 (m, 1H), 1.18-1-28 (m, 4H), 1.44 (s, 9H), 1.90-1.94 (dd, *J* = 4Hz, *J* = 8Hz, 1H), 4.03-4.06 (m, 2H), 4.11-4.16 (m2H), 4.75 (bs, 1H); ^{13}C NMR ($CDCl_3$, 100 MHz) δ 14.2, 14.9, 22.3, 27.0, 28.2, 54.0, 61.0, 64.4, 82.2, 156.8, 170.8, 171.4. MS-ESI (*m/z*) 322.0 $[M+Na]^+$, 298.2 $[M-H]^-$. HRMS-ESI (*m/z*) calcd for $C_{14}H_{22}NO_6$ $[M + H]^+$ 300.1442, found 300.1447 (Δ=1.61 ppm).

(1*R*,3*R*,4*S*)-1-(Ethoxycarbonyl)-5-azaspiro[2.3]hexane-4-carboxylic acid (27a): A solution of **(1*R*,3*R*,4*S*)-5-(*tert*-butoxycarbonyl)-1-(ethoxycarbonyl)-5-azaspiro[2.3]hexane-4-carboxylic acid** (**26a**, 0.013 g, 0.043 mmol) in formic acid (0.5 mL) was stirred at room temperature for 24 h. The crude was concentrated in vacuum to afford compound **27a** as a pale yellow solid. Precipitation from MeOH afforded the desired product as a white solid in 55% yield (0.006 g). $[\alpha]^{20}_D$ = + 3.4 (c=0.58, MeOH). ^1H NMR (400 MHz, MeOD, 25°C): *δ:* 1.19-1.23 (dd, *J* = 4Hz, *J* = 8Hz, 1H), 1.27 (t, *J* = 8 Hz, 3H), 1.53 (t, *J* = 4Hz 1H), 1.96-2.00 (dd, *J* = 4Hz, *J* = 8Hz, 1H), 3.98 (d, *J* = 12Hz, 1H), 4.16-4.21 (q, *J* = 8 Hz, 2H), 4.26 (d, *J* = 12Hz, 1H), 4.53 (bs, 1H); ^{13}C NMR ($CDCl_3$, 100 MHz, 25°C) δ: 13.2, 13.9, 23.5, 29.2, 51.2, 60.5,

63.6, 169.6, 170.8. MS-ESI (*m/z*) 200.1 [M+H]$^+$, 222.0 [M+Na]$^+$. HRMS-ESI (*m/z*) calcd for $C_9H_{14}NO_4$ [M + H]$^+$ 200.0917, found 200.0921 (Δ=1.83 ppm).

(1*S*,3*S*,4*S*)-1-(Ethoxycarbonyl)-5-azaspiro[2.3]hexane-4-carboxylic acid (27c): Compound **27c** was synthesized following the same procedure described for compound **27a**, using as starting material (1*S*,3*S*,4*S*)-5-(*tert*-butoxycarbonyl)-1-(ethoxycarbonyl)-5-azaspiro[2.3]hexane-4-carboxylic acid (**26c**, 0.012 g, 0.040 mmol). Precipitation from MeOH afforded the desired compound as a white solid in 45% yield (0.005 g). $[\alpha]^{20}_D$ = +93.1 (c=0.44, MeOH). ^{1}H NMR (400 MHz, MeOD, 25°C): *α:* 1.16 (t, *J* = 4Hz 1H), 1,29 (t, *J* = 8 Hz, 3H), 1.56-1.60 (dd, *J* = 4Hz, *J* = 8Hz, 1H), 2.08-2.12 (dd, *J* = 4Hz, *J* = 8Hz, 1H), 4.05 (d, *J* = 12Hz, 1H), 4.16-4.23 (m, 3H), 4.70 (bs, 1H); ^{13}C NMR ($CDCl_3$, 100 MHz, 25°C) δ: 12.8, 14.9, 21.9, 29.2, 49.4, 60.7, 63.8, 168.8, 171.5. MS-ESI (*m/z*)200.1 [M+H]$^+$, 222.0 [M+Na]$^+$. HRMS-ESI (*m/z*) calcd for $C_9H_{14}NO_4$ [M + H]$^+$ 200.0917, found 200.0920 (Δ=1.23 ppm).

References

1. Payack, J. F.; Huffman, M. A.; Cai, D.; Hughes, D. L.; Collins, P. C.; Johnson, B. K.; Cottrell, I. F.; Tuma, L. D. *Org. Proc. Res. Dev.,* **2004**, *8*, 256.

2. Payack, J. F.; Hughes, D. L.; Cai, D.; Cottrell, I. F.; Verhoeven, T. R. *Org. Synth.,* **2002**, *79*, 19.

3. Chen, F. M. F.; Benoiton, N. L. *Can. J. Chem.*, **1987**, *65*, 619.

4. Cesar, M. A. J.; Štefanič, P.; Kikelj, D.; Janeš, D.; Murn, J.; Nadrah, K.; Tominc, M.; Addicks, E.; Giannis, A.; Stegnar, M.; Dolenc, M. S. *Eur. J. Org. Chem.*, **2005**, 25.

5. Hanessian, S.; Fu, J. M.; Chiara, J. J. L.; Di Fabio, R. *Tetrahedron Lett.,* **1993**, *34,* 4157.

6. Hanessian, S.; Fu, J. *Can. J. Chem.,* **2001**, *79*, 1812.

7. Tebbe, F. N.; Parshall, G. W.; Reddy, G. S. *J. Am. Chem. Soc*., **1978**, *100*, 3611.

8. Cannizzo, L. F.; Grubbs, R. H. *J. Org. Chem.,* **1985**, *50,* 2386.

9. Grubbs, R. H.; Clawson, L. C.; Buchwald, S.L. *Tetrahedron Lett.,* **1984**, *25,* 5733.

10. Pine, S. H.; Shen, G. S.; Hoang, H. *Synthesis,* **1991**, 165.

11. Martínez, S.; Howell, A. R. *Tetrahedron Lett.,* **2000**, *41*, 5607.

12. Petasis, N.A.; Bzowrj, E. I. *J. Am. Chem. Soc.,* **1990**, *112*, 6392.

13. Doyle, M. P.; Griffin, J. H.; Bagheri, V.; Dorow, R. L. *Organomet.,* **1984**, *3*, 53.

14. Doyle, M. P.; Dorow, R. L.; Buhro, W. E.; Griffin, J. H.; Tamblyn, W. H.; Trudell, M. L. *Organomet.,* **1984**, *3*, 44.

15. Zhang, J. L.; Che, C. M. *Org. Lett.,* **2002**, *4*, 1911.

16. Uchida, T.; Katsuki, T. *Synthesis,* **2006**, *10*, 1715.
