# Supplementary material for: Stereocontrolled synthesis of 5-azaspiro[2.3]hexane derivatives as conformationally “frozen” analogues of L-glutamic acid
Source: Beilstein J Org Chem. 2014 May 14;10:1114–20. doi: 10.3762/bjoc.10.110 (PMC4077356; doi:10.3762/bjoc.10.110)
Supplement: File 2 — NOe studies and HPLC analysis on compounds 20a and 20c. [file Beilstein_J_Org_Chem-10-1114-s002.pdf]

Supporting Information File 2

for

Stereocontrolled synthesis of 5-azaspiro[2.3]hexane derivatives as conformationally “frozen” analogues of L-glutamic acid

Beatrice Bechi[1,2], David Amantini[3,4], Cristina Tintori[1], Maurizio Botta*[1] and Romano di Fabio*[3,5]

Address: [1]Università degli Studi di Siena, Dipartimento Farmaco Chimico Tecnologico, Via A. Moro 2, 53100, Siena, Italy; [2]Present address: Manchester Institute of Biotechnology, School of Chemistry, University of Manchester, Oxford Road, Manchester, M13 9PL, UK, [3]Neurosciences Centre of Excellence for Drug Discovery, GlaxoSmithKline Medicines Research Centre, Via A. Fleming 4, 37135, Verona, Italy, [4]Present address: Galapagos SASU, 102 avenue Gaston Roussel, 93230 Romainville, France and [5]Present address: Drug Design and Discovery, Aptuit S.r.l., Via A. Fleming 4, 37135 Verona, Italy

Email: Maurizio Botta - botta.maurizio@gmail.com, Romano di Fabio - romano.difabio@aptuit.com

* Corresponding author

NOE studies on compounds 20a and 20c

When H1 proton of the diastereoisomer obtained in 49% yield (major diasteroisomer) was irradiated, 4.8% NOE effect was observed on H2 proton (see figure below). This is possible only for the trans isomer **20a** and cis diasteroisomer **20b**, while in the other 2 diastereoisomers **20c** and **20d** this protons are too far away to afford a NOE effect. Since this reaction proceeded with trans diastereoselectivity, the major product was identified as **20a**. This was confirmed also through irradiation of the proton H3: in this case, no NOE effect was observed both at the H2 and H3 protons, while in the case of the **20b**, it should be observed. Accordingly, when NOE experiments were performed on the second most abundant diasteroisomer obtained in 32% yield, no NOE effect was observed. Therefore, this compound was identified as the *trans*-cyclopropane derivative **20c**.

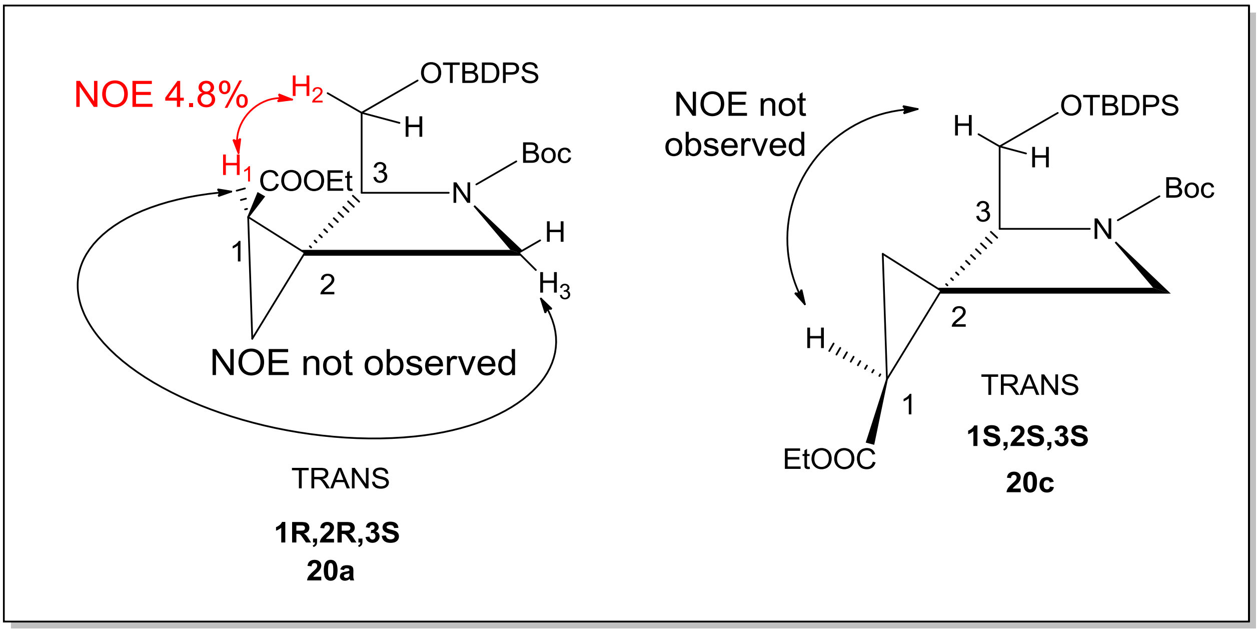

TRANS
1R,2R,3S
20a

TRANS
1S,2S,3S
20c

HPLC analysis on compound 20

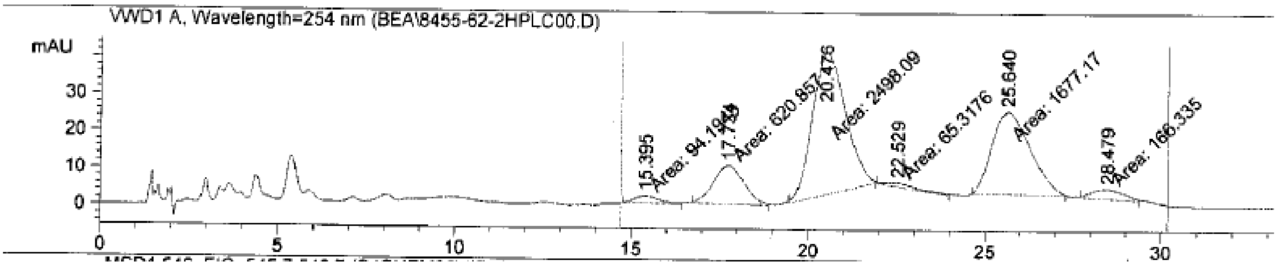

Determined by analytical HPLC–MS using an (*S*,*S*)-Whelk-O1 column (methanol/water 85:15, flow rate: 1.0 mL/min, UV-254 nm).
