# Supplementary material for: Stereocontrolled synthesis of 5-azaspiro[2.3]hexane derivatives as conformationally “frozen” analogues of L-glutamic acid
Source: Beilstein J Org Chem. 2014 May 14;10:1114–20. doi: 10.3762/bjoc.10.110 (PMC4077356; doi:10.3762/bjoc.10.110)
Supplement: File 3 — Gaussian input files for QM calculations for compounds 20a, 20b, 20c and 20d. [file Beilstein_J_Org_Chem-10-1114-s003.pdf]

Supporting Information File 3

for

Stereocontrolled synthesis of 5-azaspiro[2.3]hexane derivatives as conformationally “frozen” analogues of L-glutamic acid

Beatrice Bechi[1,2], David Amantini[3,4], Cristina Tintori[1], Maurizio Botta*[1] and Romano di Fabio*[3,5]

Address: [1]Università degli Studi di Siena, Dipartimento Farmaco Chimico Tecnologico, Via A. Moro 2, 53100, Siena, Italy; [2]Present address: Manchester Institute of Biotechnology, School of Chemistry, University of Manchester, Oxford Road, Manchester, M13 9PL, UK, [3]Neurosciences Centre of Excellence for Drug Discovery, GlaxoSmithKline Medicines Research Centre, Via A. Fleming 4, 37135, Verona, Italy, [4]Present address: Galapagos SASU, 102 avenue Gaston Roussel, 93230 Romainville, France and [5]Present address: Drug Design and Discovery, Aptuit S.r.l., Via A. Fleming 4, 37135 Verona, Italy

Email: Maurizio Botta - botta.maurizio@gmail.com, Romano di Fabio - romano.difabio@aptuit.com

* Corresponding author

Gaussian Input Files for QM calculations for compounds 20a, 20b, 20c and 20d

Compound RRS (**20a**)

C	-1.46780	-0.35790	-3.98230
C	-1.50370	-1.69950	-3.28910
N	-0.04870	-1.68220	-3.40000
C	0.04410	-0.37160	-4.04740
C	-2.39130	0.84220	-3.85560
C	0.71790	0.66130	-3.13440
O	0.11810	0.59270	-1.85330
C	-2.37800	0.01860	-5.12640
C	-3.59270	0.73620	-2.96070
O	-4.74260	0.87780	-3.38360
O	-3.25470	0.50260	-1.68250
C	-4.24860	0.34160	-0.68060
C	-4.64050	-1.13530	-0.55260
H	-1.95220	1.83430	-3.93720
C	0.91030	-2.38710	-2.68710
O	0.29290	-3.30290	-1.90190
O	2.12560	-2.19630	-2.75030
C	0.95440	-4.14770	-0.95190
C	1.89320	-5.15050	-1.65300
C	1.69260	-3.33380	0.13320
C	-0.19710	-4.92020	-0.28730
Si	0.14940	1.78340	-0.64720
C	-0.83480	3.24430	-1.36980
C	-0.76400	0.91480	0.77130
C	1.97410	2.15380	-0.12570
C	2.74000	2.91910	-1.22120
C	1.97960	3.00230	1.15900
C	2.68230	0.81220	0.14550
C	-2.12640	3.53820	-0.87880
C	-2.88620	4.59460	-1.41800
C	-2.36090	5.37480	-2.46390
C	-1.07850	5.09250	-2.96850
C	-0.32500	4.03350	-2.42510
C	-1.20150	1.63280	1.90530
C	-1.87590	0.97910	2.95540
C	-2.11330	-0.40660	2.88420
C	-1.67370	-1.13570	1.76360
C	-1.00350	-0.47580	0.71510
H	-1.85920	-1.68130	-2.25820
H	-1.97820	-2.50220	-3.85450
H	0.45340	-0.41790	-5.05770
H	1.78840	0.47290	-3.05310
H	0.60740	1.65950	-3.55530
H	-1.92310	0.46920	-6.00580

H	-3.83620	0.69130	0.26600
H	-5.12350	0.96060	-0.88490
H	-5.36860	-1.27210	0.24670
H	-5.08470	-1.50990	-1.47500
H	-3.77270	-1.75290	-0.32120
H	2.29860	-5.87050	-0.94130
H	1.36790	-5.71400	-2.42480
H	2.74330	-4.65820	-2.12480
H	1.02820	-2.61960	0.61920
H	2.53790	-2.77550	-0.26830
H	2.08840	-3.98840	0.91020
H	0.17250	-5.61380	0.46880
H	-0.89860	-4.24440	0.20370
H	-0.76100	-5.50220	-1.01730
H	3.79070	3.04130	-0.95610
H	2.70920	2.40630	-2.18100
H	2.33470	3.92050	-1.36410
H	2.99420	3.26020	1.46440
H	1.42990	3.93450	1.02070
H	1.51780	2.47120	1.99180
H	3.71470	0.95840	0.46440
H	2.17470	0.24590	0.92790
H	2.69920	0.18270	-0.74530
H	-2.54980	2.93890	-0.08590
H	-3.87410	4.80340	-1.03070
H	-2.94200	6.18600	-2.88080
H	-0.67280	5.68640	-3.77620
H	0.65170	3.82560	-2.83490
H	-1.01980	2.69720	1.96580
H	-2.20970	1.54130	3.81680
H	-2.63010	-0.91020	3.68950
H	-1.85260	-2.20050	1.70540
H	-0.67360	-1.04130	-0.14490

Compound SRS (**20b**)

C	-1.27360	-1.04720	-1.01930
C	0.09100	-1.56730	-0.63450
N	0.53210	-1.05460	-1.92390
C	-0.73900	-0.45730	-2.31690
C	-2.46070	-0.67670	-0.14140
C	-0.59530	1.07040	-2.35150
O	-0.27400	1.53850	-1.05400

C	-2.49090	-1.94070	-0.97540
C	-3.33920	0.47530	-0.55470
O	-3.90270	0.53350	-1.65150
O	-3.40440	1.42030	0.39260
C	-4.14190	2.60680	0.14710
C	-4.07860	3.50900	1.38170
H	-2.29750	-0.78480	0.92890
C	1.75970	-1.08670	-2.56220
O	2.66390	-1.68690	-1.75190
O	1.98390	-0.63490	-3.68550
C	4.04920	-1.87530	-2.06940
C	4.22820	-2.81070	-3.28310
C	4.78110	-0.53130	-2.26960
C	4.62170	-2.56670	-0.82080
Si	0.31070	3.08460	-0.67380
C	-0.66760	4.28210	-1.78390
C	-0.13510	3.24990	1.16230
C	2.23510	3.12090	-0.86170
C	2.67930	2.86110	-2.31250
C	2.76890	4.49460	-0.41720
C	2.82920	2.02190	0.03960
C	-0.46260	4.32910	-3.18110
C	-1.20740	5.20630	-3.99390
C	-2.17620	6.04780	-3.41730
C	-2.39970	6.00580	-2.02910
C	-1.65070	5.12700	-1.22240
C	-0.06300	4.49760	1.81900
C	-0.38900	4.61270	3.18480
C	-0.78450	3.47390	3.91150
C	-0.84840	2.22200	3.27150
C	-0.52090	2.11320	1.90570
H	0.55290	-1.05740	0.21220
H	0.16870	-2.65080	-0.53580
H	-1.15320	-0.89370	-3.22720
H	0.14850	1.38100	-3.08370
H	-1.53240	1.53270	-2.66350
H	-3.12640	-1.96710	-1.85880
H	-2.40750	-2.88520	-0.44200
H	-3.72930	3.12560	-0.71930
H	-5.17940	2.35930	-0.08210
H	-4.63810	4.42960	1.21620
H	-4.50190	3.01230	2.25480
H	-3.05090	3.78330	1.61750
H	5.28030	-3.05090	-3.44010
H	3.69570	-3.75170	-3.14170
H	3.86540	-2.36160	-4.20730
H	5.85340	-0.68480	-2.39470
H	4.43370	0.00390	-3.15290

H	4.64510	0.12640	-1.41150
H	5.68690	-2.77210	-0.93230
H	4.49940	-1.94720	0.06860
H	4.12370	-3.51770	-0.62770
H	3.76600	2.81120	-2.38880
H	2.29200	1.91730	-2.69080
H	2.35420	3.65540	-2.98260
H	3.85110	4.56060	-0.53350
H	2.32450	5.30040	-1.00340
H	2.54420	4.69140	0.63130
H	3.91800	2.00410	-0.01190
H	2.55630	2.17230	1.08500
H	2.46640	1.03420	-0.24920
H	0.26470	3.67990	-3.64400
H	-1.03750	5.23000	-5.06180
H	-2.75020	6.72070	-4.03950
H	-3.14790	6.64590	-1.58190
H	-1.84240	5.09940	-0.15960
H	0.24100	5.37570	1.26570
H	-0.33510	5.57520	3.67480
H	-1.03590	3.56010	4.95970
H	-1.14870	1.34430	3.82710
H	-0.57040	1.14850	1.42030

Compound SSS (**20c**)

C	-0.26130	-1.52930	2.12210
C	-1.05200	-2.72020	1.63000
N	-0.43900	-2.46380	0.33580
C	0.34890	-1.30070	0.75380
C	-0.72770	-0.42750	3.06040
C	1.85990	-1.55120	0.66400
O	2.18610	-2.79640	1.25300
C	0.39150	-1.36280	3.47340
C	-2.06480	-0.56560	3.74110
O	-2.43590	-1.60120	4.30140
O	-2.78920	0.55930	3.64640
C	-4.08390	0.60990	4.22530
C	-4.69330	1.99140	3.97440
H	-0.44380	0.58460	2.77920
C	-0.42800	-3.24870	-0.80690
O	0.40020	-2.69760	-1.72430
O	-1.07560	-4.28590	-0.95310

C	0.77010	-3.30420	-2.96820
C	-0.43750	-3.41440	-3.92080
C	1.79120	-2.32030	-3.56400
C	1.46260	-4.66750	-2.75760
Si	3.69240	-3.57360	1.14050
C	3.20990	-5.37610	0.79760
C	4.63110	-2.76990	-0.31080
C	4.56630	-3.41820	2.85290
C	3.66700	-4.06830	3.92100
C	5.92790	-4.13470	2.81590
C	4.78030	-1.93790	3.21870
C	1.85560	-5.77260	0.84200
C	1.48510	-7.10630	0.58110
C	2.47200	-8.06310	0.27890
C	3.82680	-7.68330	0.24090
C	4.19080	-6.34730	0.50110
C	5.00310	-1.40680	-0.28300
C	5.68490	-0.81860	-1.36640
C	6.00710	-1.59090	-2.49720
C	5.64540	-2.94960	-2.54030
C	4.96320	-3.53110	-1.45360
H	-0.76230	-3.67570	2.06940
H	-2.13600	-2.60020	1.62590
H	0.02420	-0.37660	0.27300
H	2.19390	-1.51970	-0.37290
H	2.40260	-0.76010	1.18220
H	0.18460	-2.12430	4.22310
H	1.39640	-0.94860	3.50570
H	-4.71710	-0.16420	3.78920
H	-4.02010	0.41690	5.29720
H	-5.69040	2.05780	4.40980
H	-4.08270	2.77760	4.41890
H	-4.78160	2.19460	2.90700
H	-0.12740	-3.76170	-4.90690
H	-1.18570	-4.11840	-3.55730
H	-0.92850	-2.44980	-4.05310
H	2.16420	-2.66760	-4.52800
H	1.35220	-1.33390	-3.71870
H	2.65290	-2.19390	-2.90730
H	1.86850	-5.05110	-3.69390
H	2.28850	-4.58700	-2.05150
H	0.77920	-5.42520	-2.37490
H	4.11040	-4.00370	4.91510
H	2.69040	-3.58340	3.96270
H	3.49430	-5.12410	3.70740
H	6.46140	-4.02810	3.76100
H	5.81310	-5.20240	2.62800
H	6.56700	-3.73090	2.02920

H	5.16440	-1.83060	4.23360
H	5.50210	-1.46150	2.55640
H	3.84930	-1.37320	3.16070
H	1.09190	-5.04490	1.07660
H	0.44310	-7.39380	0.61330
H	2.19030	-9.08760	0.07830
H	4.58770	-8.41640	0.01070
H	5.23300	-6.06120	0.46550
H	4.75230	-0.79660	0.57180
H	5.95830	0.22710	-1.33030
H	6.52910	-1.14160	-3.33090
H	5.88800	-3.54680	-3.40860
H	4.68580	-4.57370	-1.50730

Compound RSS (**20d**)

C	-4.50160	0.59690	0.72220
C	-5.18370	-0.62950	0.17540
N	-3.93680	-1.33330	0.45340
C	-3.23360	-0.17940	1.01150
C	-4.59520	2.01040	0.17430
C	-2.94810	-0.34110	2.50400
O	-2.04530	-1.40650	2.73020
C	-5.14390	1.68190	1.55380
C	-3.32130	2.81190	0.04470
O	-2.88910	3.16160	-1.05550
O	-2.71570	3.05440	1.22070
C	-1.47590	3.73990	1.25410
C	-1.00160	3.82050	2.70680
H	-5.34940	2.20930	-0.58400
C	-3.44020	-2.44710	-0.20800
O	-2.09320	-2.51340	-0.05680
O	-4.12720	-3.23250	-0.86280
C	-1.24890	-3.49140	-0.68200
C	-1.57730	-4.92000	-0.20080
C	-1.29980	-3.38270	-2.22080
C	0.17060	-3.12630	-0.21720
Si	-0.72040	-1.31190	3.79070
C	0.05950	-3.02780	3.67560
C	0.39250	0.01920	3.01880
C	-1.34890	-0.96310	5.58750
C	-1.80390	0.50140	5.74600
C	-2.54090	-1.89270	5.88570

C	-0.22820	-1.26690	6.60090
C	1.45780	-3.18770	3.77510
C	2.03990	-4.46880	3.70270
C	1.22330	-5.60390	3.53840
C	-0.17390	-5.45590	3.45080
C	-0.75090	-4.17240	3.52240
C	0.40930	0.17340	1.61590
C	1.24020	1.12960	1.00050
C	2.07650	1.94180	1.78820
C	2.08340	1.78840	3.18710
C	1.25030	0.82840	3.79560
H	-6.02730	-0.98980	0.76540
H	-5.43790	-0.57900	-0.88460
H	-2.37300	0.12420	0.41410
H	-2.53850	0.59270	2.89120
H	-3.86240	-0.53900	3.06520
H	-6.22640	1.65380	1.65790
H	-4.61330	2.02510	2.43950
H	-0.74060	3.21030	0.64700
H	-1.59040	4.74090	0.83610
H	-0.03650	4.32300	2.77100
H	-1.70980	4.37410	3.32320
H	-0.88680	2.82540	3.13660
H	-0.84330	-5.63680	-0.57010
H	-1.57540	-4.98200	0.88670
H	-2.55270	-5.26010	-0.54760
H	-0.56210	-4.03880	-2.68390
H	-2.27130	-3.66680	-2.62450
H	-1.08810	-2.36630	-2.55450
H	0.91110	-3.82140	-0.61390
H	0.45470	-2.12660	-0.54670
H	0.25300	-3.14980	0.86890
H	-2.67440	0.72630	5.13170
H	-2.08290	0.71870	6.77750
H	-1.01740	1.20350	5.46980
H	-2.93190	-1.73410	6.89120
H	-3.36140	-1.72920	5.18650
H	-2.25550	-2.94270	5.80810
H	-0.56540	-1.11380	7.62650
H	0.10780	-2.30200	6.52190
H	0.64510	-0.63460	6.45170
H	2.08730	-2.31740	3.90270
H	3.11320	-4.58030	3.77410
H	1.66830	-6.58780	3.48230
H	-0.80380	-6.32610	3.32690
H	-1.82420	-4.05840	3.45230
H	-0.22250	-0.45690	1.00710
H	1.23750	1.23680	-0.07550

H	2.71570	2.67750	1.32000
H	2.72950	2.40660	3.79520
H	1.27190	0.72110	4.86880
