# Supplementary material for: Stereocontrolled synthesis of 5-azaspiro[2.3]hexane derivatives as conformationally “frozen” analogues of L-glutamic acid
Source: Beilstein J Org Chem. 2014 May 14;10:1114–20. doi: 10.3762/bjoc.10.110 (PMC4077356; doi:10.3762/bjoc.10.110)
Supplement: File 4 — Copies of 1H and 13C NMR spectra for all new compounds. [file Beilstein_J_Org_Chem-10-1114-s004.pdf]

Supporting Information File 4

for

Stereocontrolled synthesis of 5-azaspiro[2.3]hexane derivatives as conformationally “frozen” analogues of L-glutamic acid

Beatrice Bechi[1,2], David Amantini[3,4], Cristina Tintori[1], Maurizio Botta*[1] and Romano di Fabio*[3,5]

Address: [1]Università degli Studi di Siena, Dipartimento Farmaco Chimico Tecnologico, Via A. Moro 2, 53100, Siena, Italy; [2]Present address: Manchester Institute of Biotechnology, School of Chemistry, University of Manchester, Oxford Road, Manchester, M13 9PL, UK, [3]Neurosciences Centre of Excellence for Drug Discovery, GlaxoSmithKline Medicines Research Centre, Via A. Fleming 4, 37135, Verona, Italy, [4]Present address: Galapagos SASU, 102 avenue Gaston Roussel, 93230 Romainville, France and [5]Present address: Drug Design and Discovery, Aptuit S.r.l., Via A. Fleming 4, 37135 Verona, Italy

Email: Maurizio Botta - botta.maurizio@gmail.com, Romano di Fabio - romano.difabio@aptuit.com

* Corresponding author

Copies of ^{1}H and ^{13}C NMR spectra of all new compounds

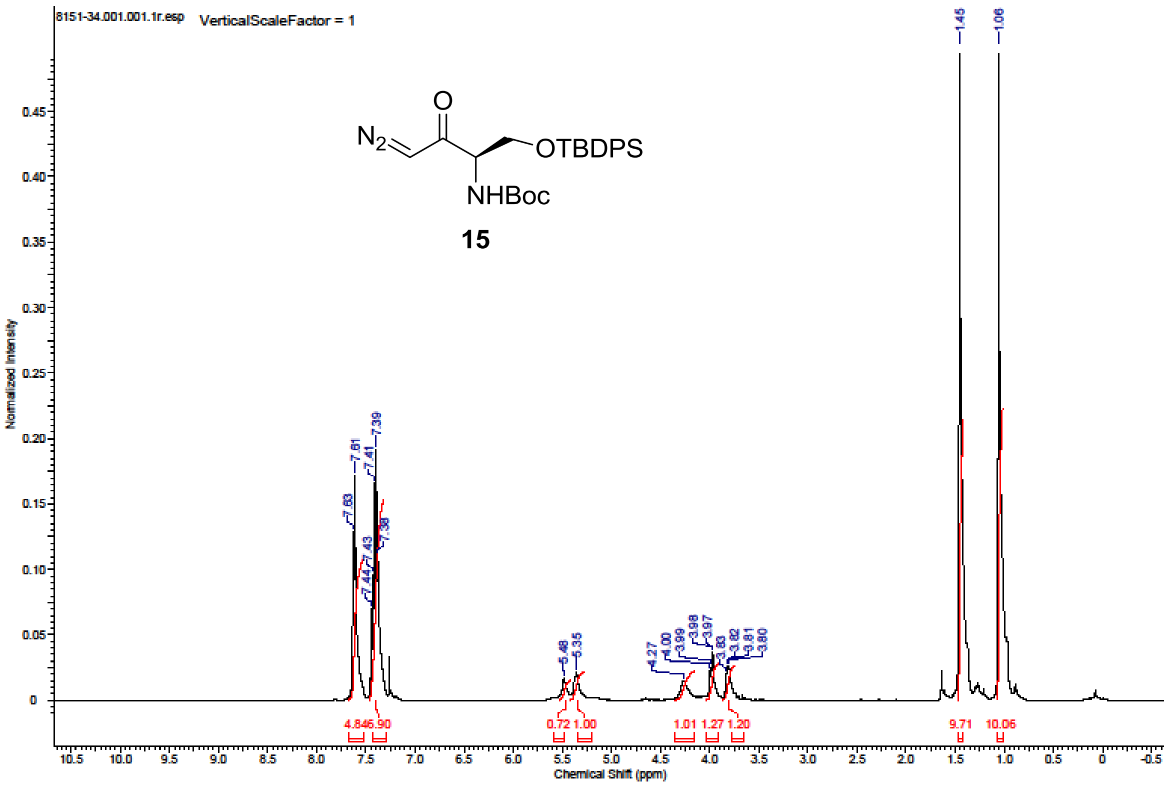
8151-34.001.001.1r.esp
VerticalScaleFactor = 1
N2
O
OTBDPS
NHBoc
15
Normalized Intensity
Chemical Shift (ppm)
4.84
6.90
0.72
1.00
1.01
1.27
1.20
9.71
10.06

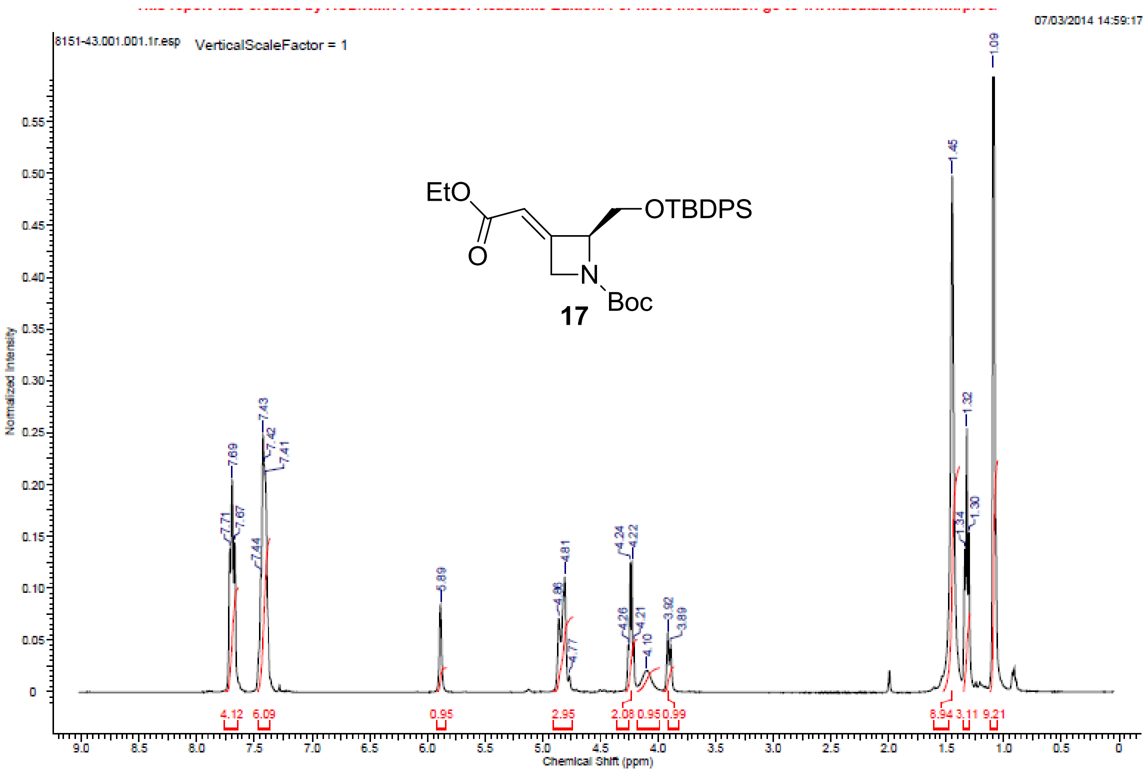
07/03/2014 14:59:17
8151-43.001.001.1r.esp
VerticalScaleFactor = 1
EtO
O
OTBDPS
N
Boc
17
Normalized Intensity
Chemical Shift (ppm)
4.12
6.09
0.95
2.95
2.08
0.95
0.99
8.94
3.11
9.21

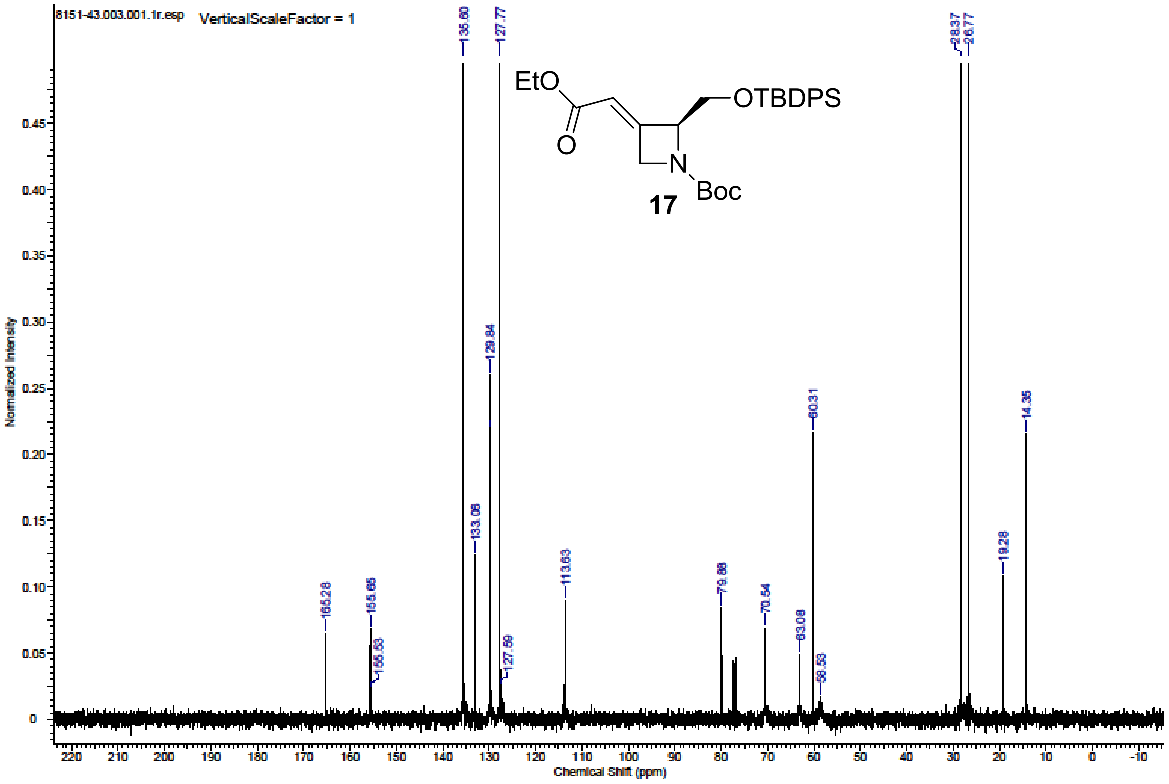

8151-43.003.001.1r.esp
VerticalScaleFactor = 1
EtO
O
OTBDPS
N
Boc
17
135.60
127.77
129.84
133.08
113.63
165.28
155.65
155.53
127.59
79.88
70.54
63.08
60.31
58.53
28.37
26.77
19.28
14.35
Normalized Intensity
Chemical Shift (ppm)

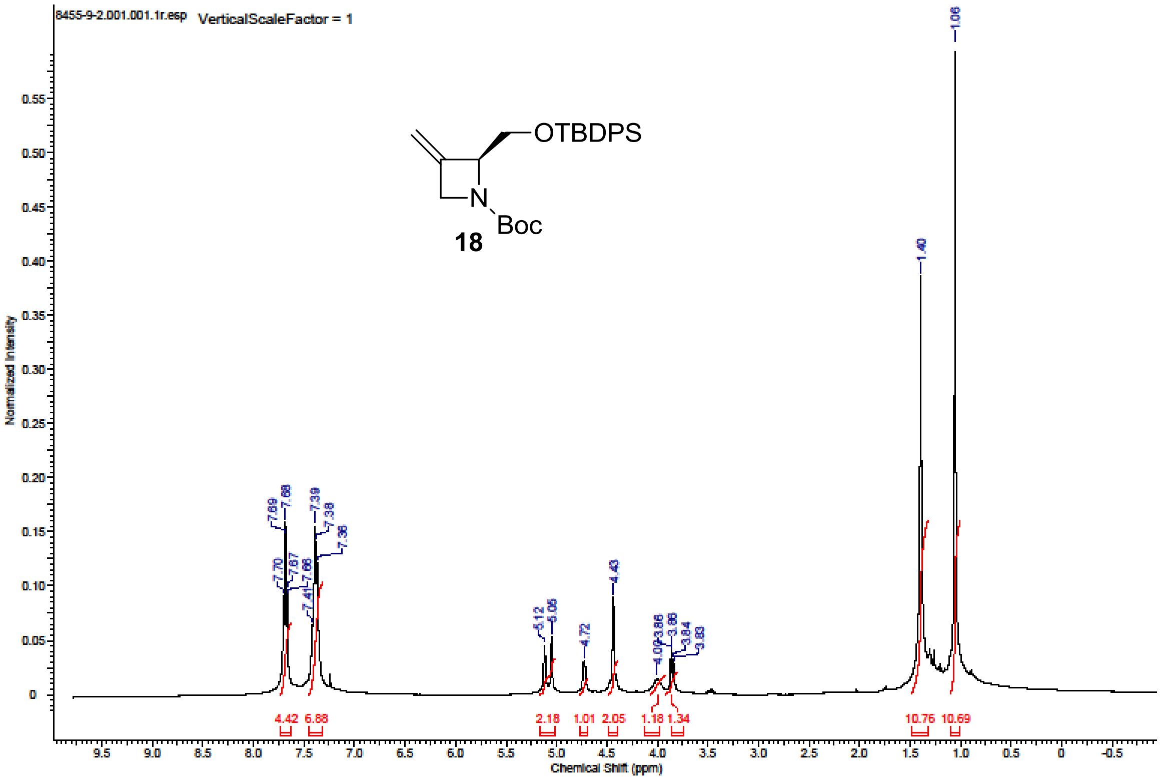
8455-9-2.001.001.1r.esp VerticalScaleFactor = 1
OTBDPS
N
Boc
18
Normalized Intensity
Chemical Shift (ppm)
4.42 6.88 2.18 1.01 2.05 1.18 1.34 10.76 10.69

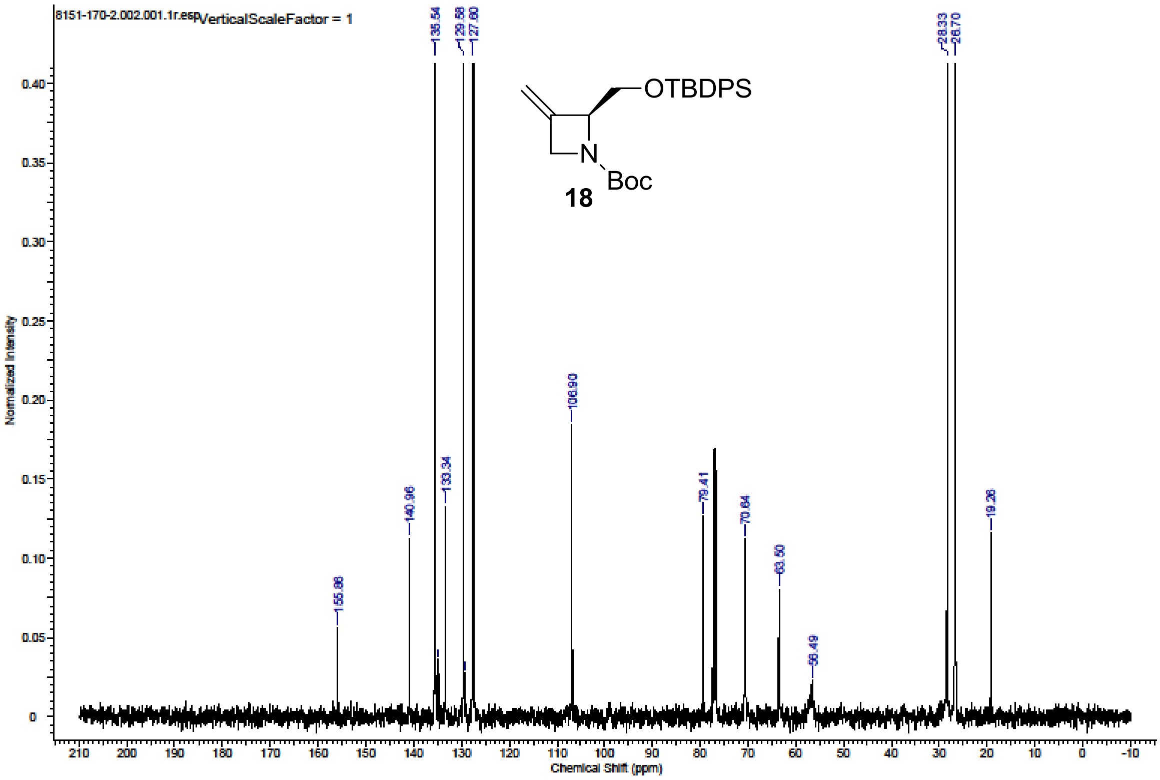

8151-170-2.002.001.1r.esp
VerticalScaleFactor = 1
OTBDPS
N
Boc
18
135.54
129.58
127.60
28.33
26.70
106.90
140.96
133.34
155.88
79.41
70.64
63.50
56.49
19.26
Normalized Intensity
Chemical Shift (ppm)

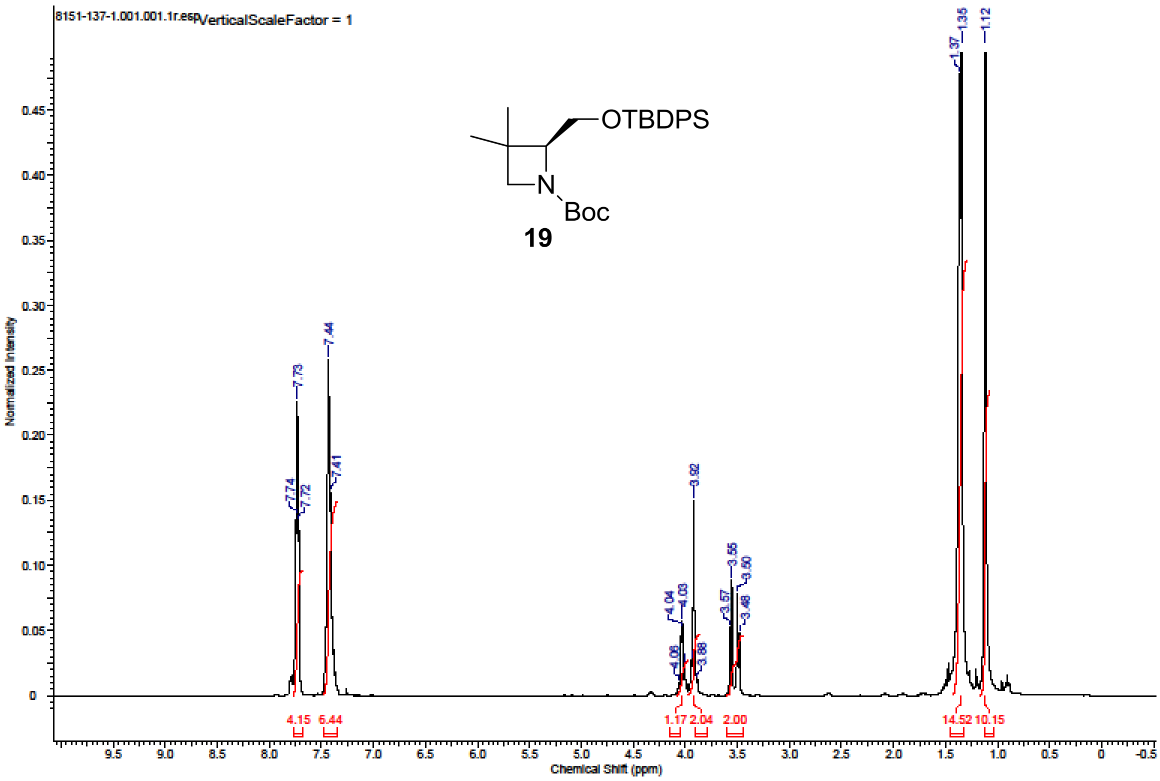
8151-137-1.001.001.1r.esp
VerticalScaleFactor = 1
OTBDPS
N
Boc
19
Normalized Intensity
Chemical Shift (ppm)
7.74
7.73
7.72
7.44
7.41
4.06
4.04
4.03
3.92
3.88
3.57
3.55
3.50
3.48
1.37
1.35
1.12
4.15
6.44
1.17
2.04
2.00
14.52
10.15

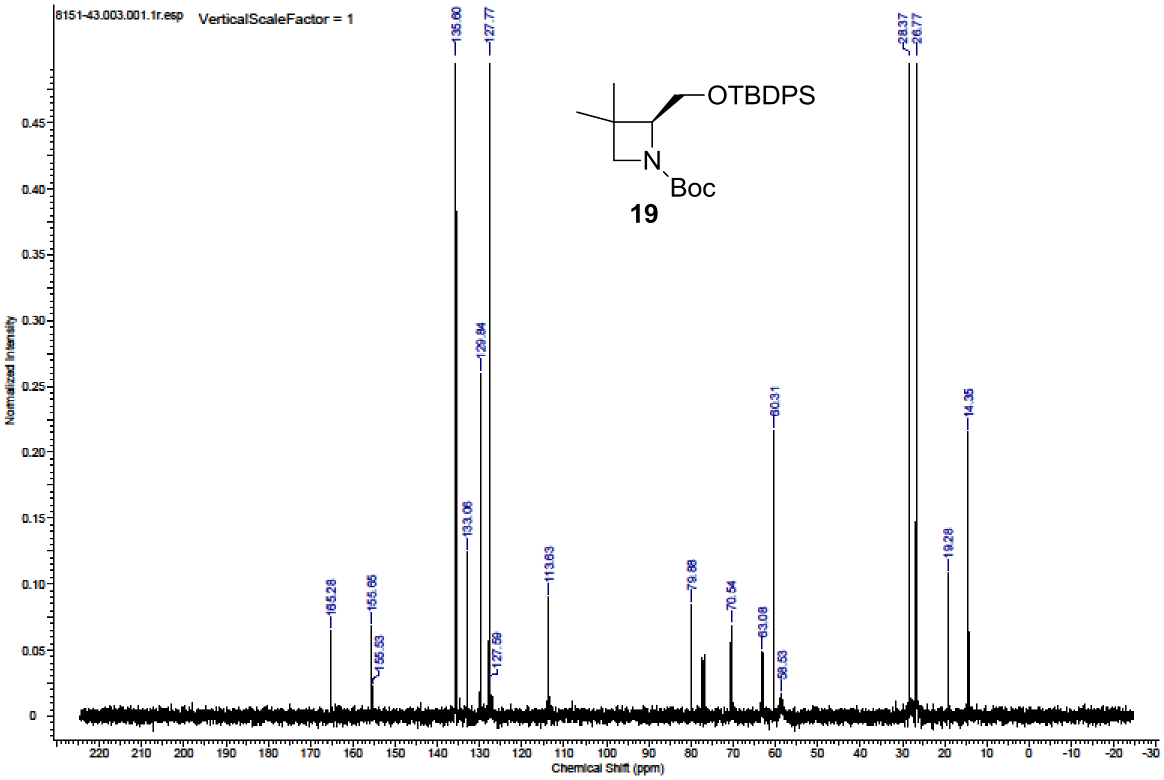
8151-43.003.001.1r.esp VerticalScaleFactor = 1
135.60
127.77
28.37
26.77
OTBDPS
N
Boc
19
129.84
60.31
14.35
133.06
113.63
19.28
165.28
155.65
79.88
70.54
63.08
155.53
127.59
58.53
Normalized Intensity
Chemical Shift (ppm)

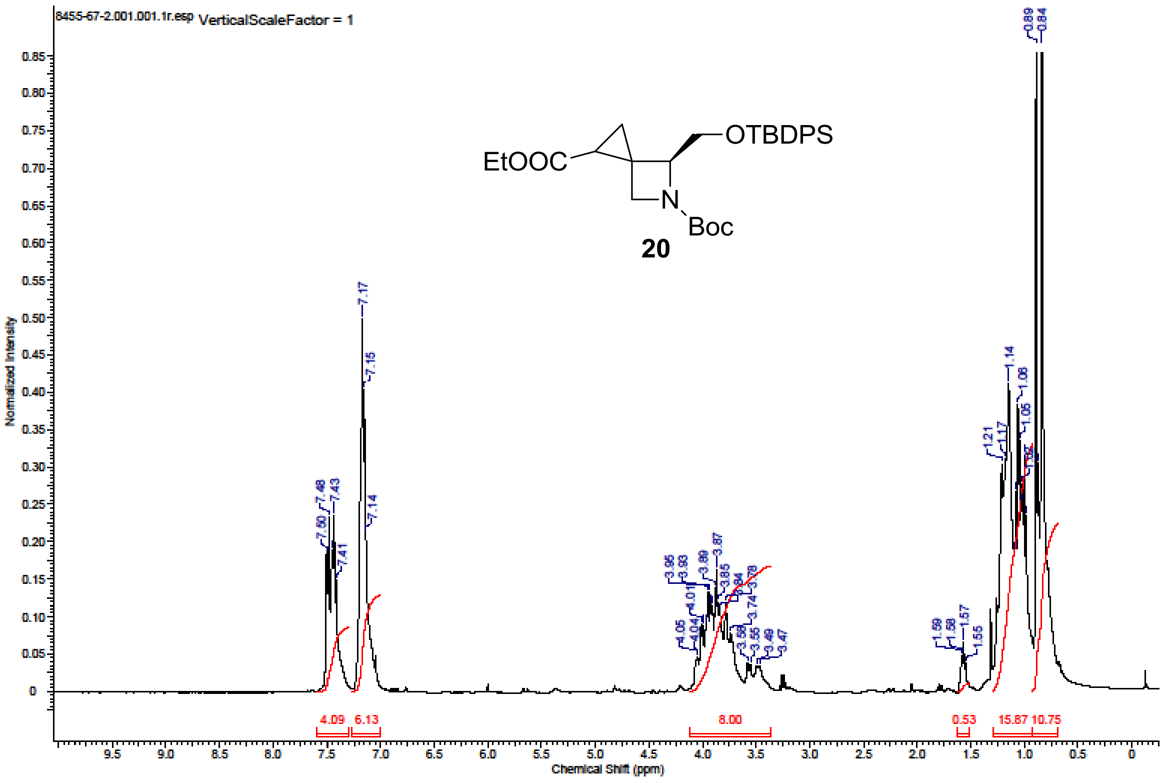
8455-67-2.001.001.1r.esp VerticalScaleFactor = 1
EtOOC
OTBDPS
N
Boc
20
Normalized Intensity
Chemical Shift (ppm)

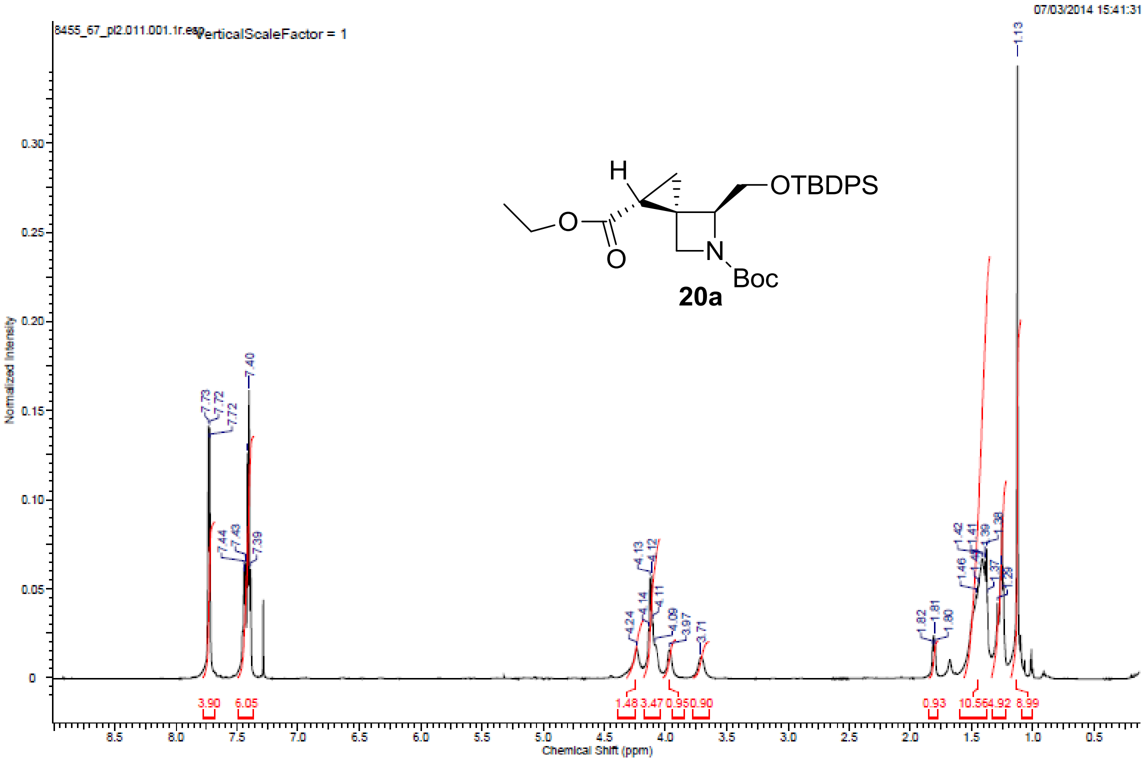
07/03/2014 15:41:31
8455_67_pl2.011.001.1r.esp VerticalScaleFactor = 1
H
O
O
OTBDPS
N
Boc
20a
Normalized Intensity
Chemical Shift (ppm)

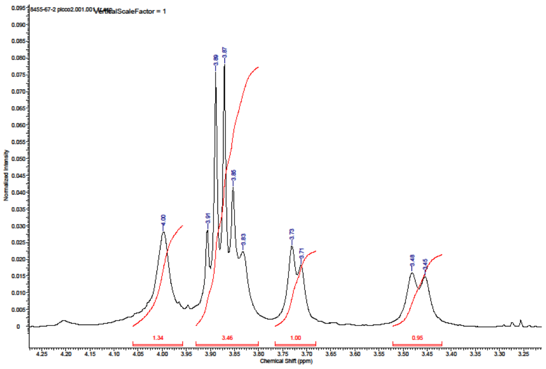

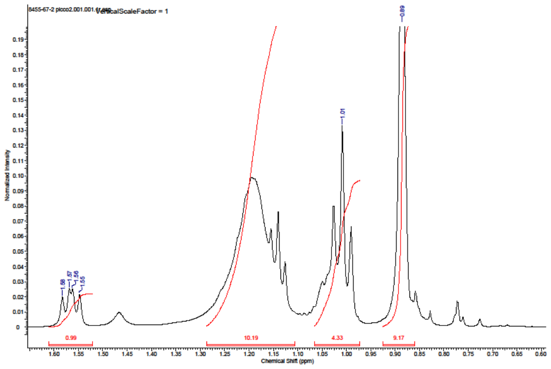

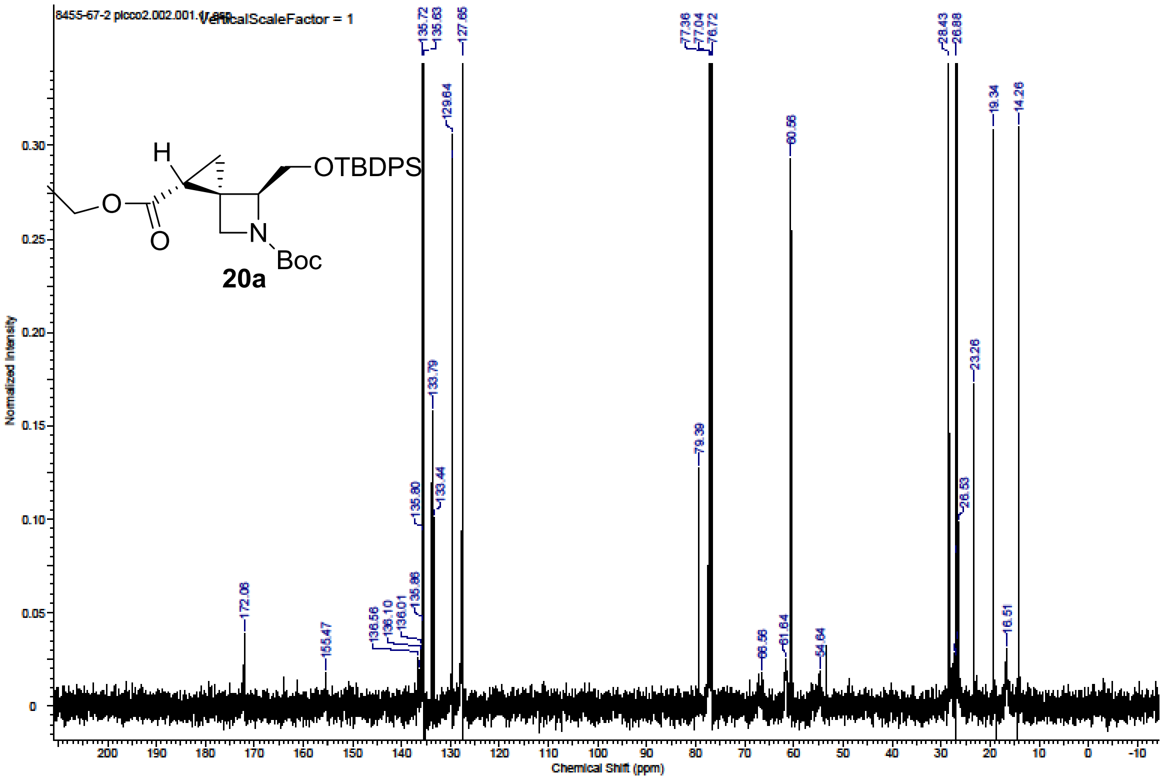
8455-67-2 picco2.002.001.1r.esp VerticalScaleFactor = 1
OTBDPS
N
Boc
20a
Normalized Intensity
Chemical Shift (ppm)

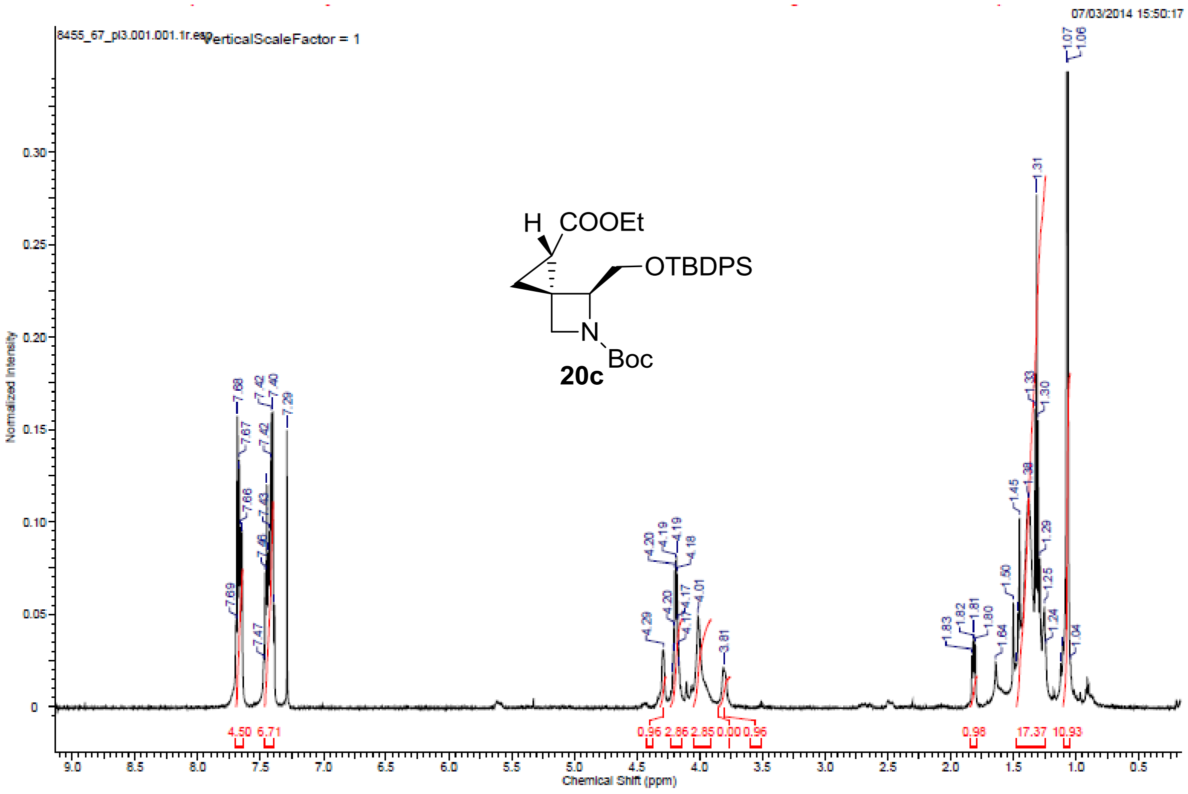
8455_67_pl3.001.001.1r.esp
VerticalScaleFactor = 1
07/03/2014 15:50:17
Normalized Intensity
Chemical Shift (ppm)
H
COOEt
OTBDPS
N
Boc
20c
4.50
6.71
0.96
2.86
2.85
0.00
0.96
0.98
17.37
10.93

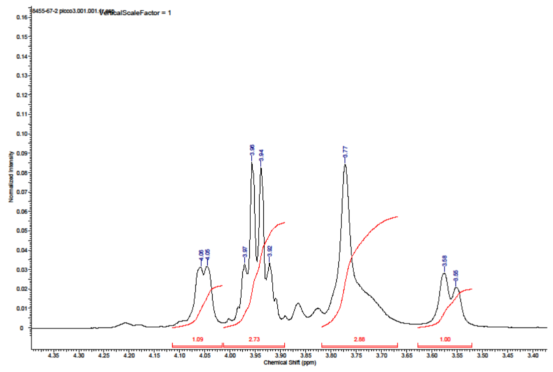
VerticalScaleFactor = 1
Normalized Intensity
Chemical Shift (ppm)

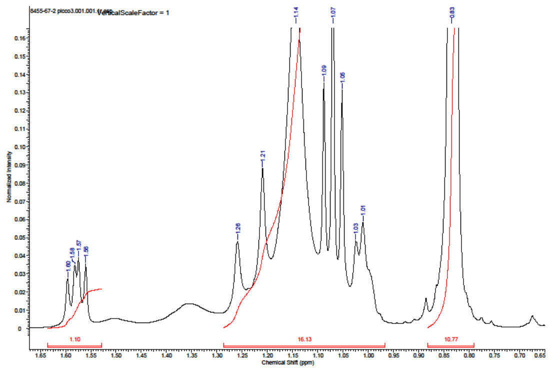
VerticalScaleFactor = 1
Normalized Intensity
Chemical Shift (ppm)

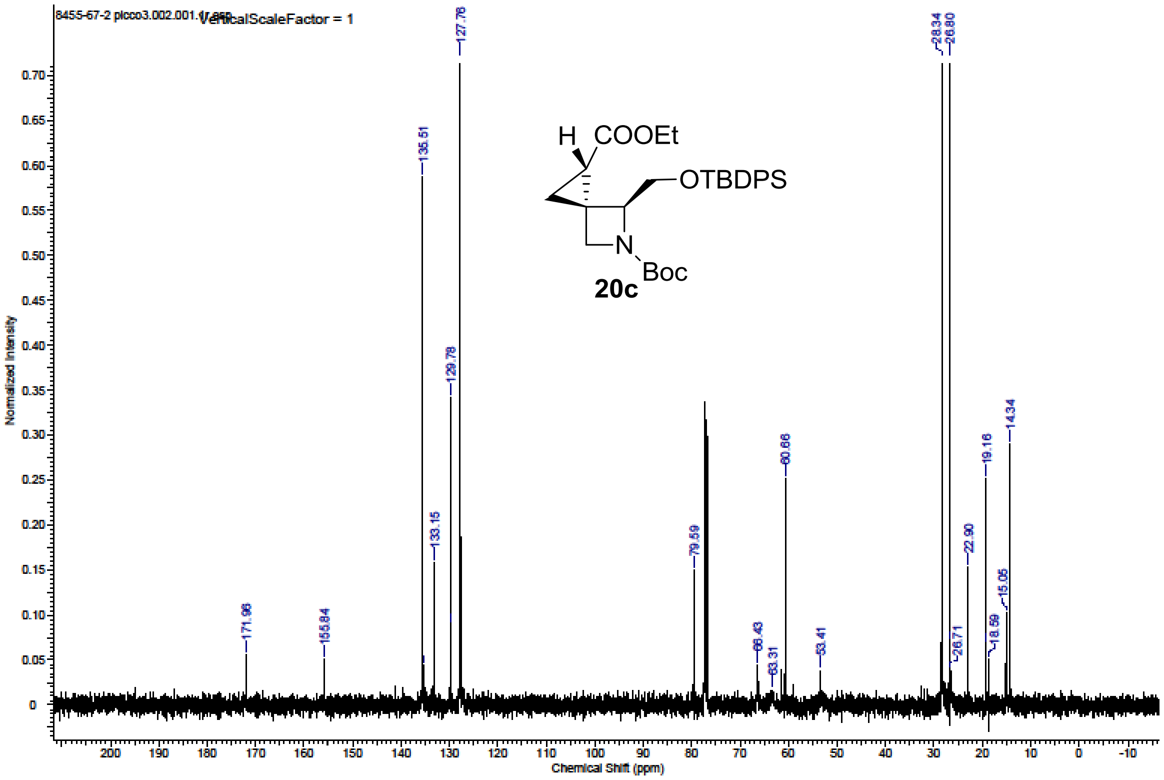
8455-67-2 plcco3.002.001.1r.esp VerticalScaleFactor = 1
H COOEt
OTBDPS
N
Boc
20c
Normalized Intensity
Chemical Shift (ppm)

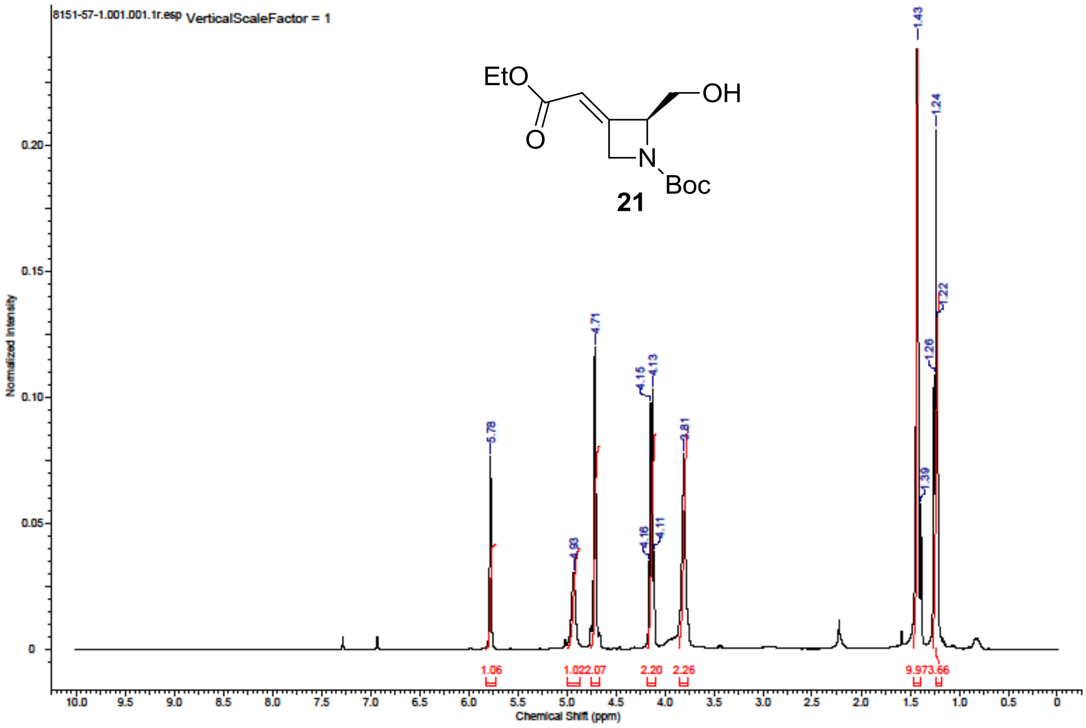
8151-57-1.001.001.1r.esp VerticalScaleFactor = 1
EtO
O
OH
N
Boc
21
Normalized Intensity
Chemical Shift (ppm)

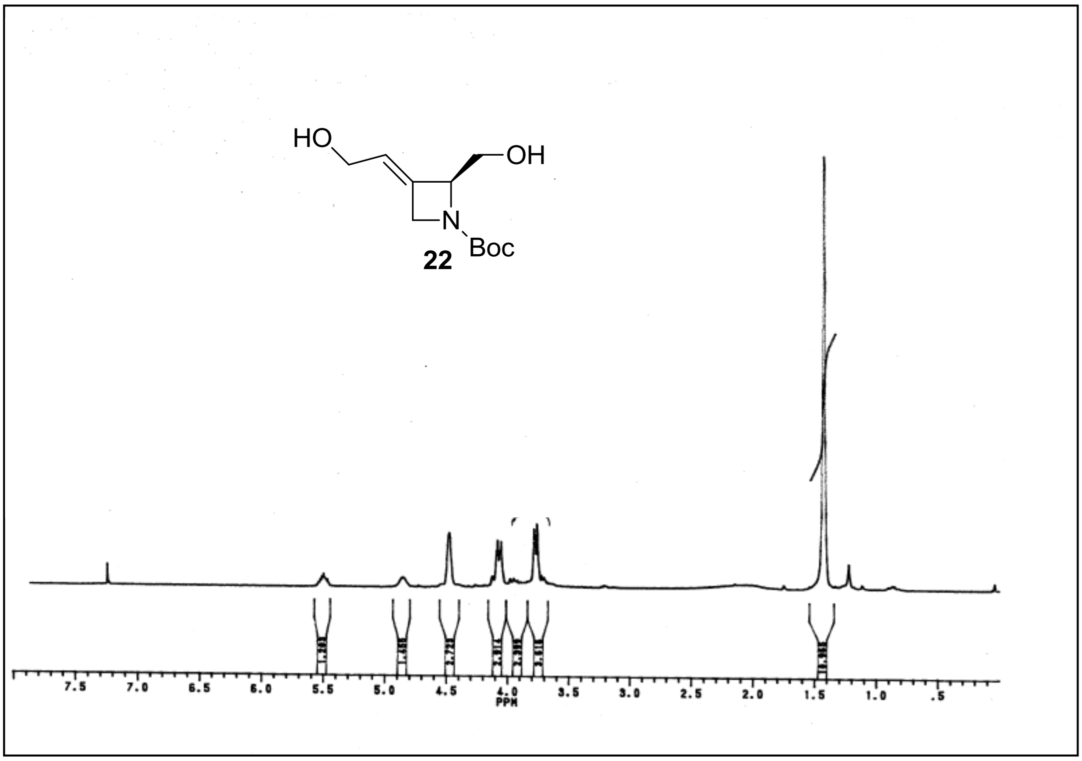
HO
OH
N
Boc
22
7.5
7.0
6.5
6.0
5.5
5.0
4.5
4.0
PPM
3.5
3.0
2.5
2.0
1.5
1.0
.5

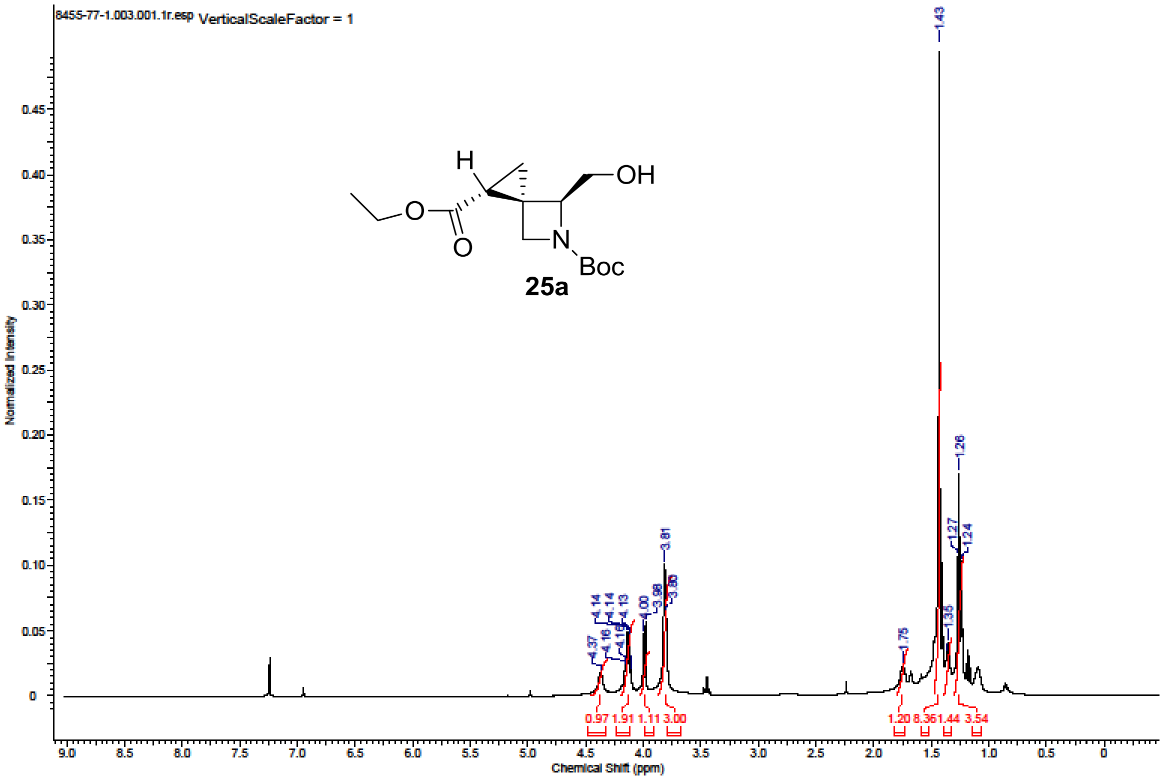

8455-77-1.003.001.1r.esp VerticalScaleFactor = 1
25a
Boc
OH
Normalized Intensity
Chemical Shift (ppm)

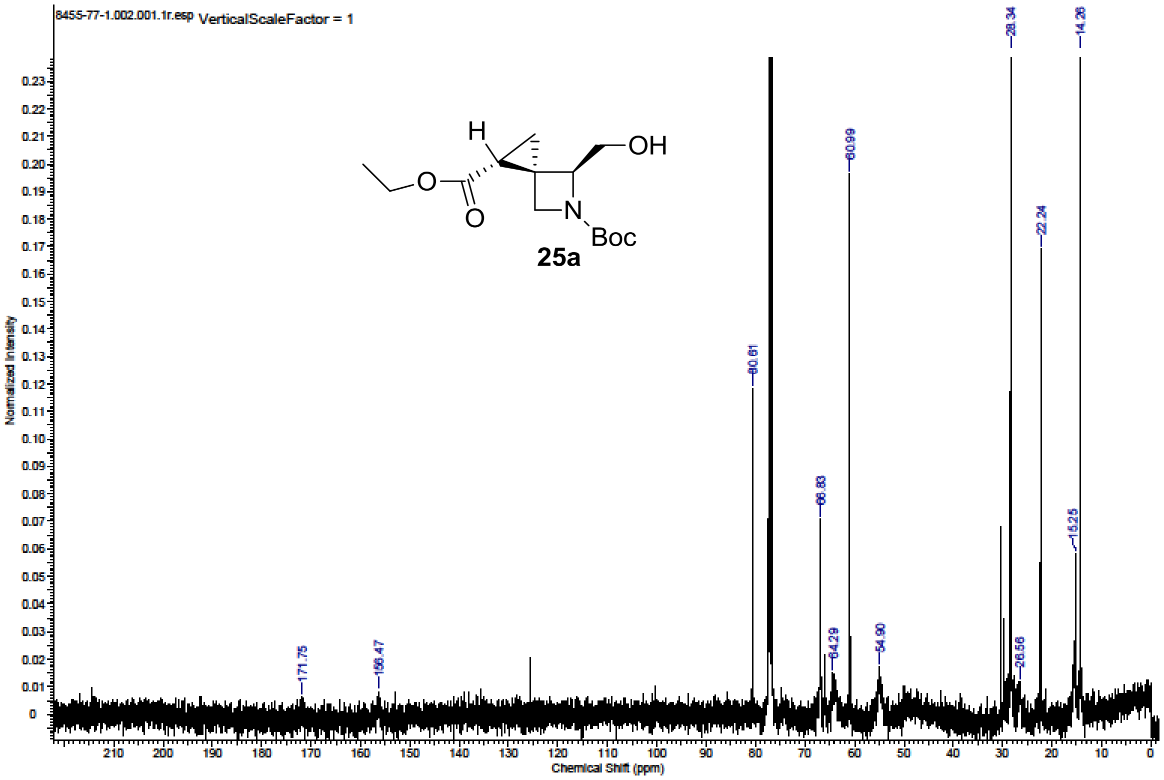
8455-77-1.002.001.1r.esp VerticalScaleFactor = 1
H
OH
O
O
N
Boc
25a
Normalized Intensity
Chemical Shift (ppm)
171.75
156.47
80.61
66.83
64.29
60.99
54.90
28.34
26.56
22.24
15.25
14.26

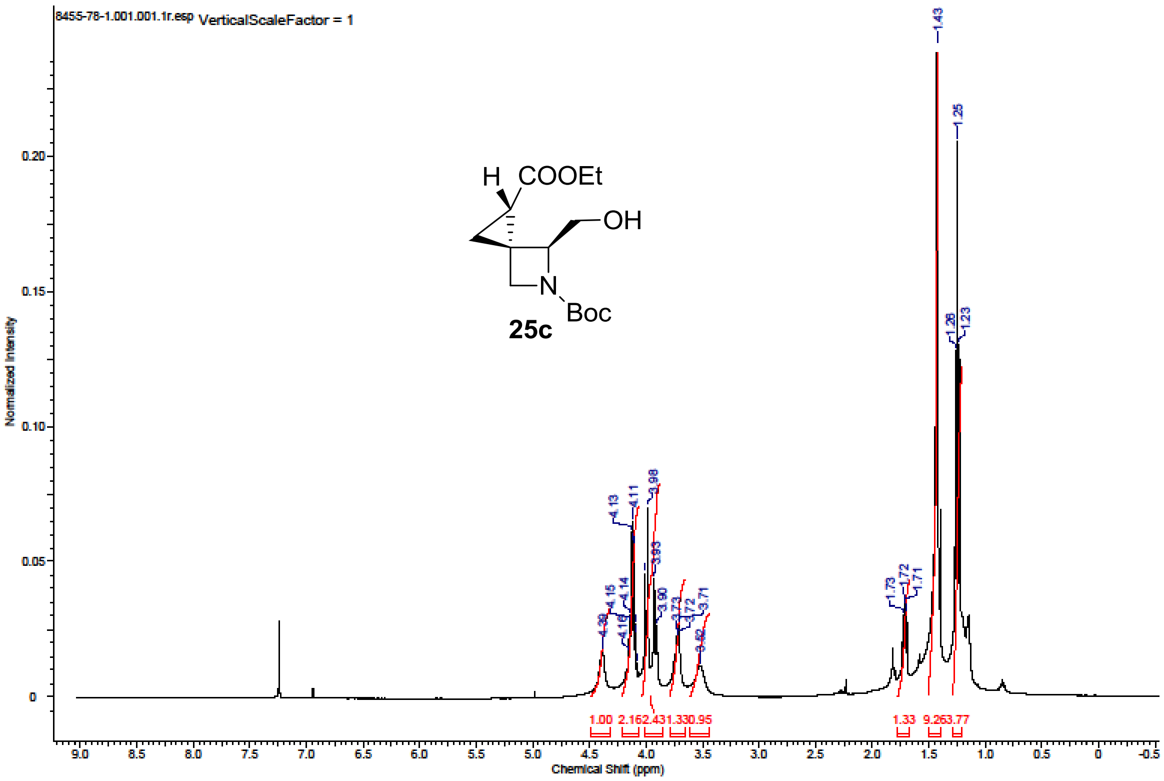
8455-78-1.001.001.1r.esp VerticalScaleFactor = 1
H COOEt
OH
N
Boc
25c
Normalized Intensity
Chemical Shift (ppm)
1.00 2.16 2.43 1.33 0.95
1.33 9.26 3.77

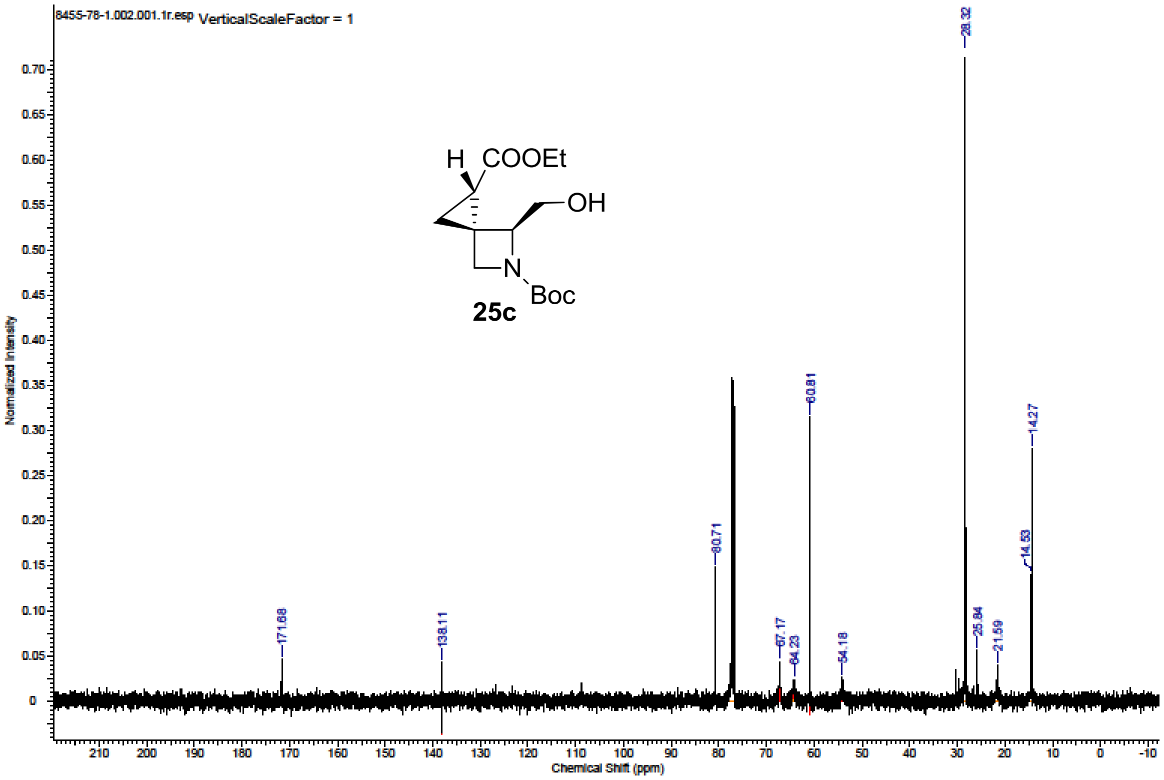
8455-78-1.002.001.1r.esp VerticalScaleFactor = 1
H COOEt
OH
N
Boc
25c
171.68
138.11
80.71
67.17
64.23
60.81
54.18
28.32
25.84
21.59
14.53
14.27
Normalized Intensity
Chemical Shift (ppm)

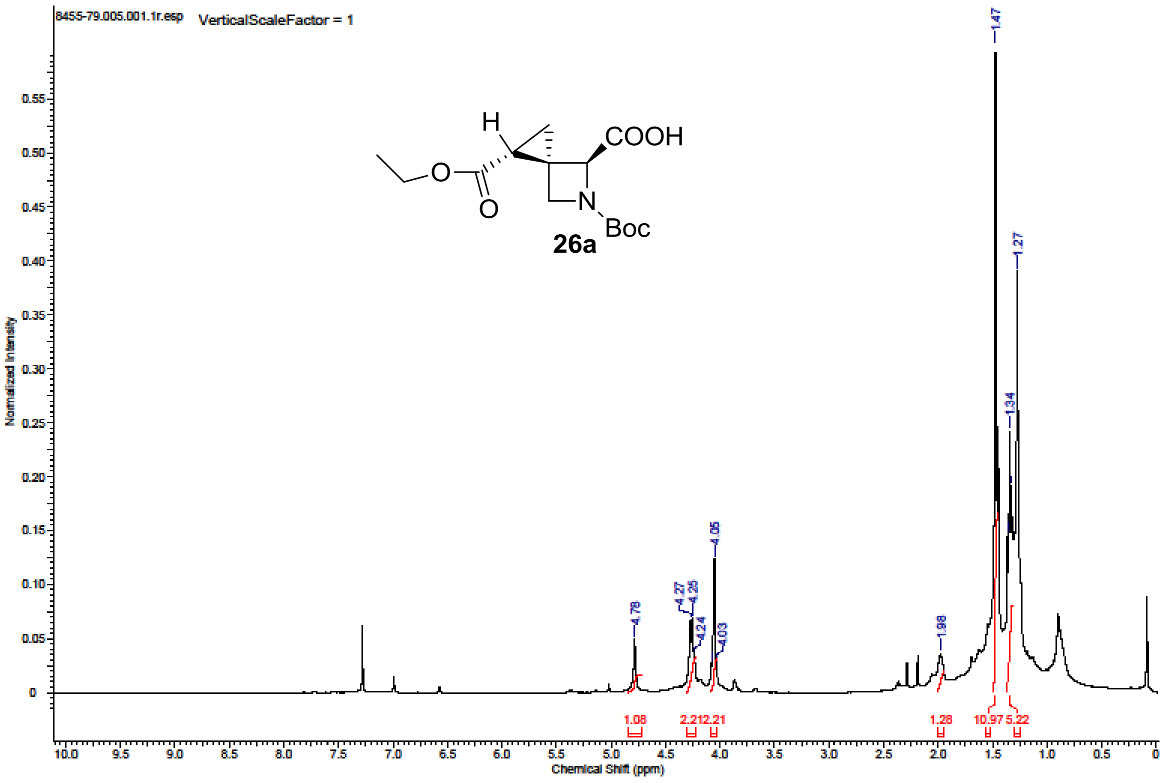
8455-79.005.001.1r.esp
VerticalScaleFactor = 1
COOH
H
O
O
N
Boc
26a
Normalized Intensity
Chemical Shift (ppm)
4.78
4.27
4.25
4.24
4.05
4.03
1.98
1.47
1.34
1.27
1.08
2.21
2.21
1.28
10.97
5.22

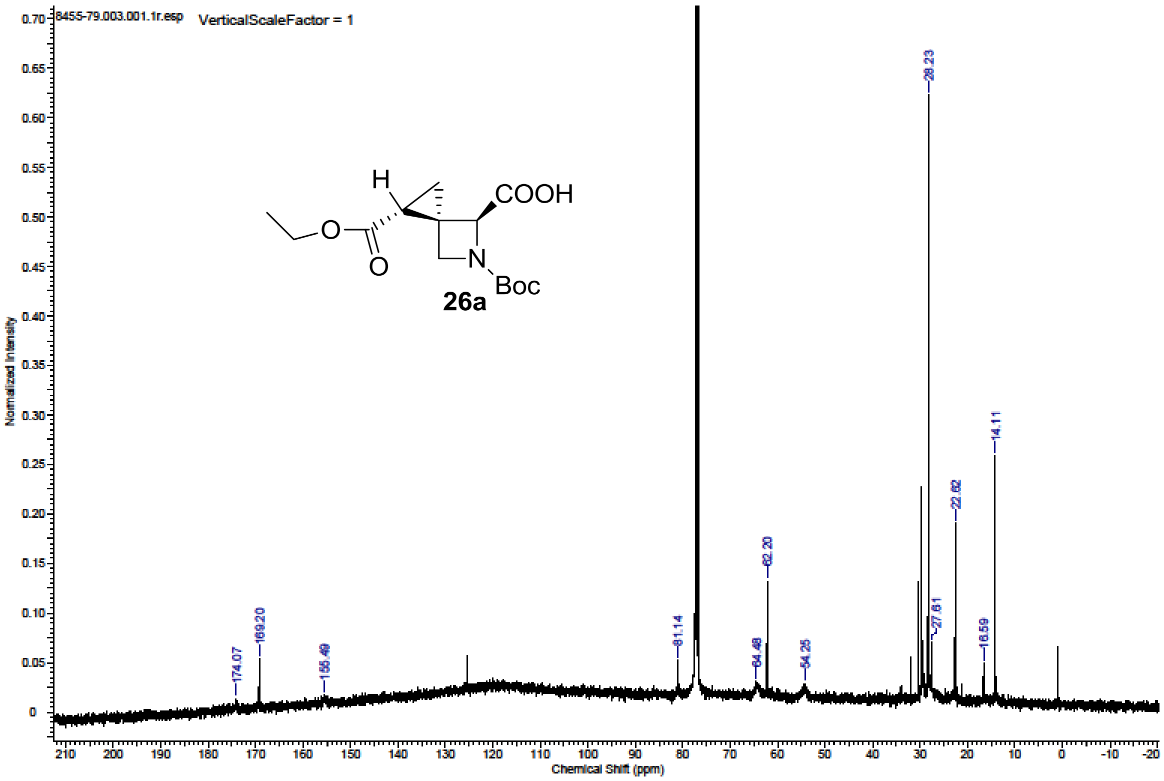
8455-79.003.001.1r.esp
VerticalScaleFactor = 1
COOH
H
O
O
N
Boc
26a
174.07
169.20
155.49
81.14
64.48
62.20
54.25
28.23
27.61
22.62
16.59
14.11
Normalized Intensity
Chemical Shift (ppm)

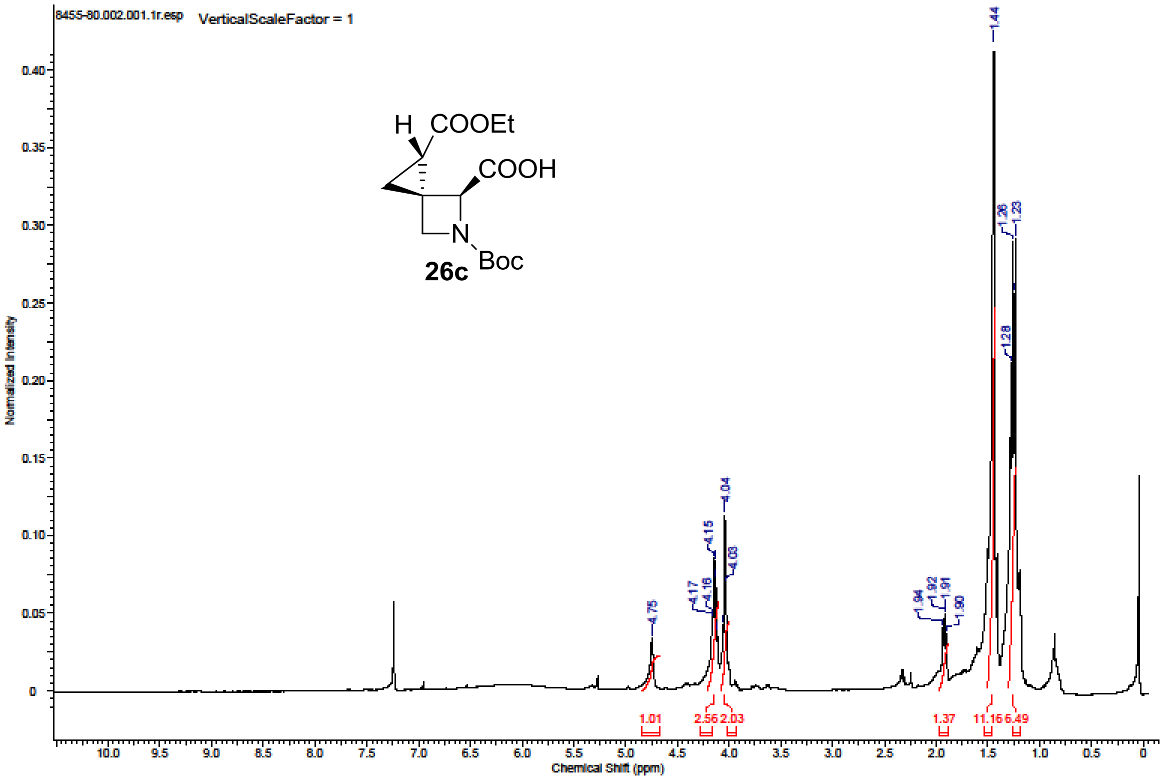
8455-80.002.001.1r.esp
VerticalScaleFactor = 1
H
COOEt
COOH
N
Boc
26c
Normalized Intensity
Chemical Shift (ppm)
1.01
2.56
2.03
1.37
11.16
6.49

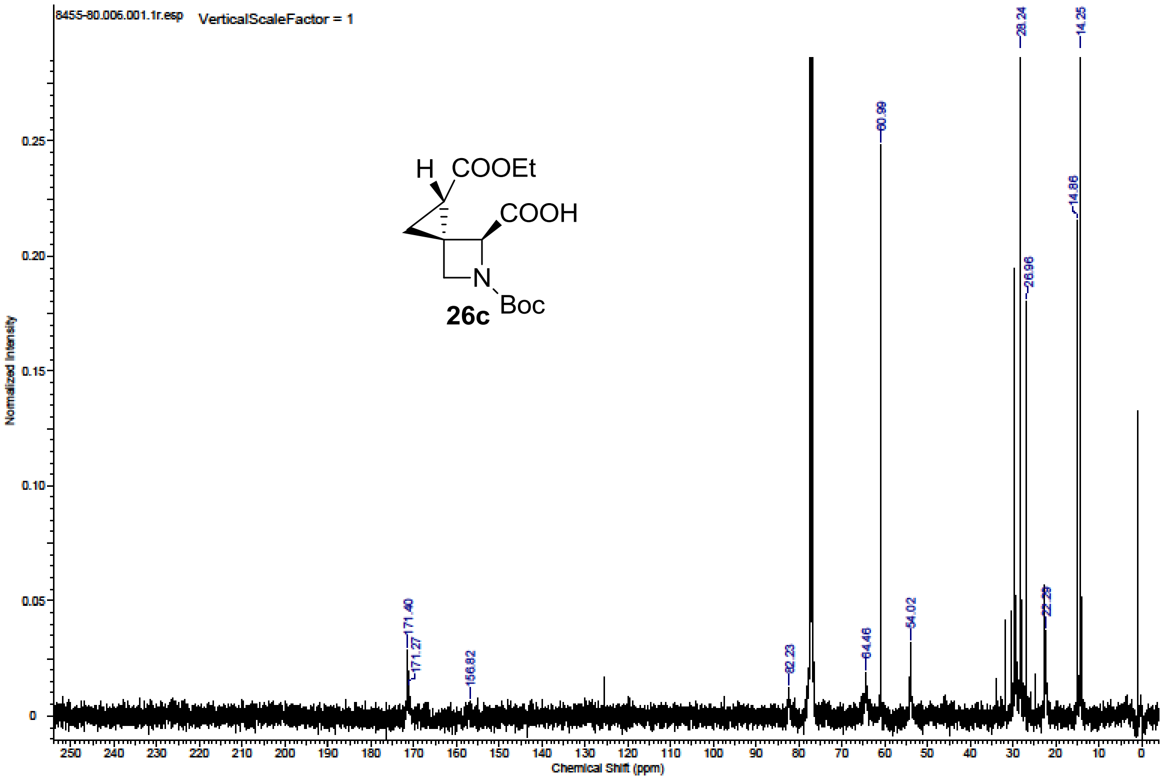
8455-80.006.001.1r.esp
VerticalScaleFactor = 1
H COOEt
COOH
N
Boc
26c
Normalized Intensity
Chemical Shift (ppm)
171.40
171.27
156.82
82.23
64.46
60.99
54.02
28.24
26.96
22.23
14.86
14.25

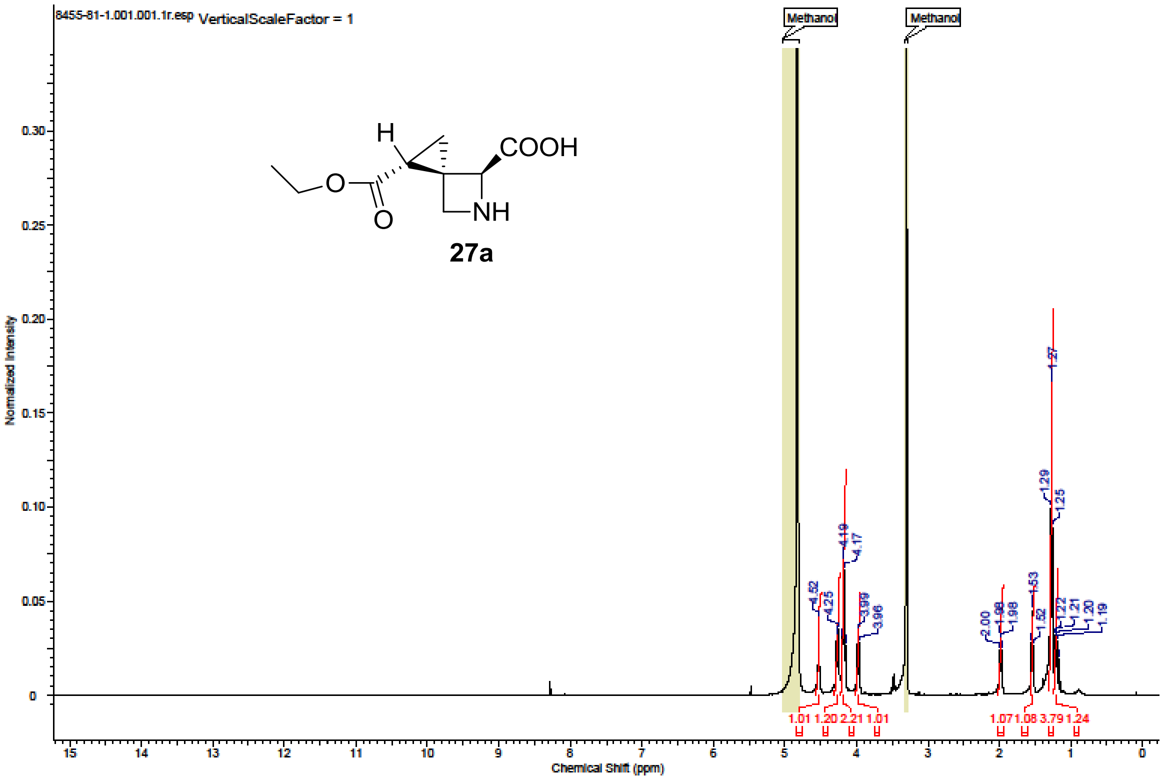
8455-81-1.001.001.1r.esp VerticalScaleFactor = 1
Methanol
Methanol
COOH
NH
H
O
O
27a
Normalized Intensity
Chemical Shift (ppm)

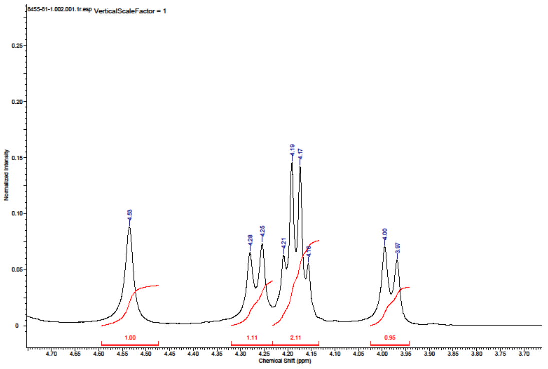

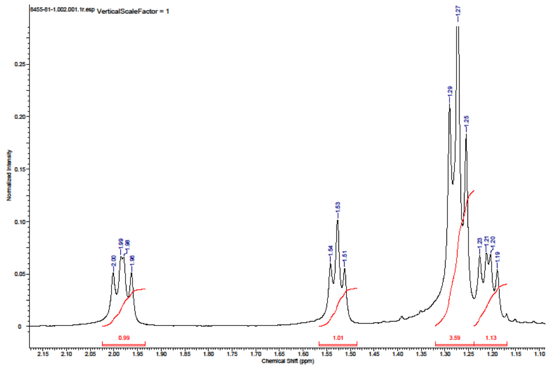

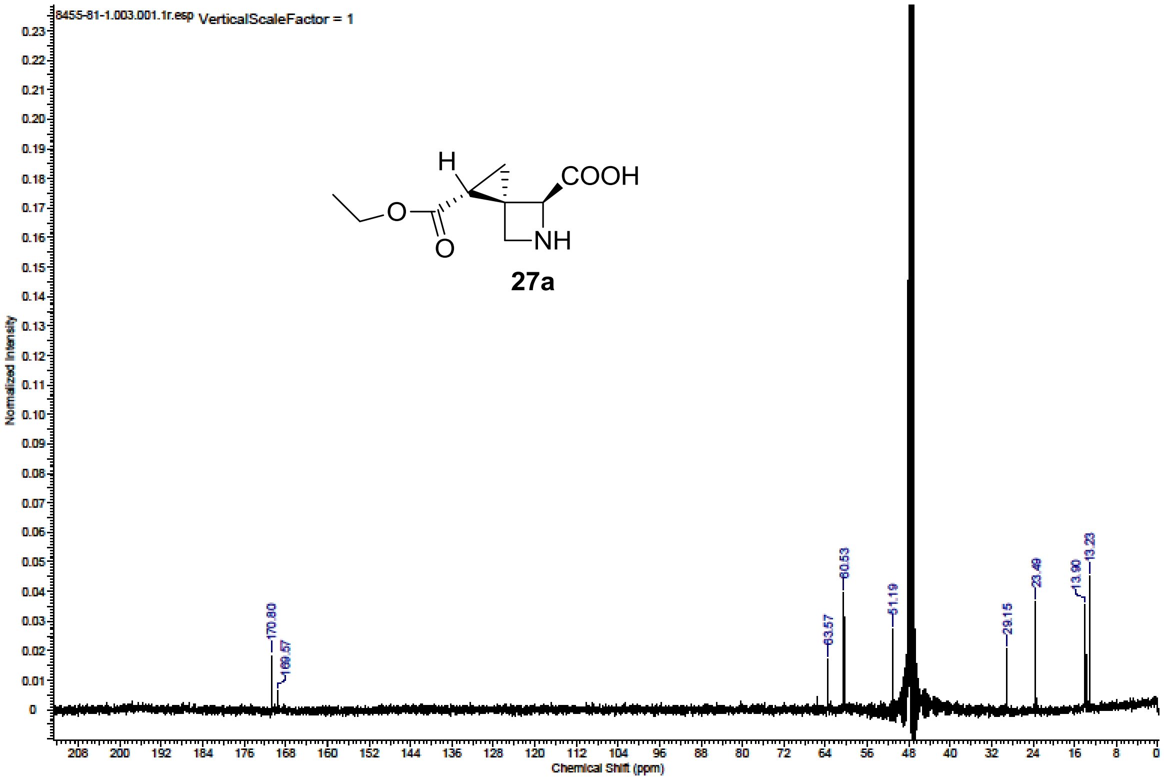
8455-81-1.003.001.1r.esp VerticalScaleFactor = 1
H
COOH
O
O
NH
27a
170.80
169.57
63.57
60.53
51.19
29.15
23.49
13.90
13.23
Normalized Intensity
Chemical Shift (ppm)

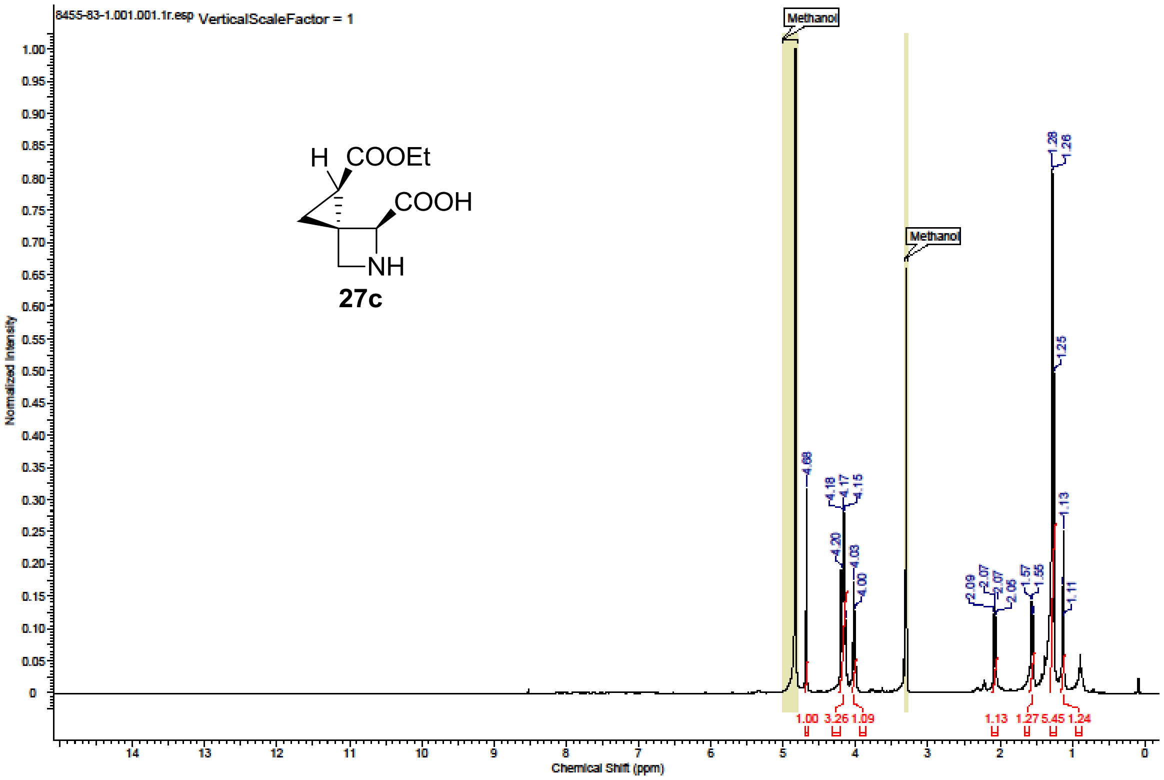

8455-83-1.001.001.1r.esp VerticalScaleFactor = 1
H COOEt
COOH
NH
27c
Methanol
Methanol
Normalized Intensity
Chemical Shift (ppm)
1.00
3.26
1.09
1.13
1.27
5.45
1.24

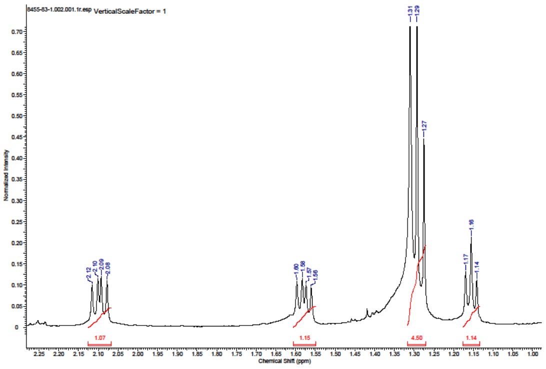

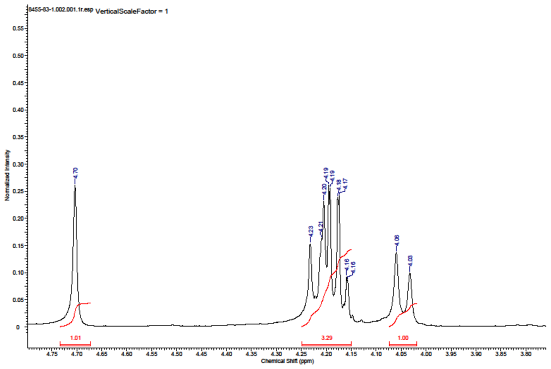

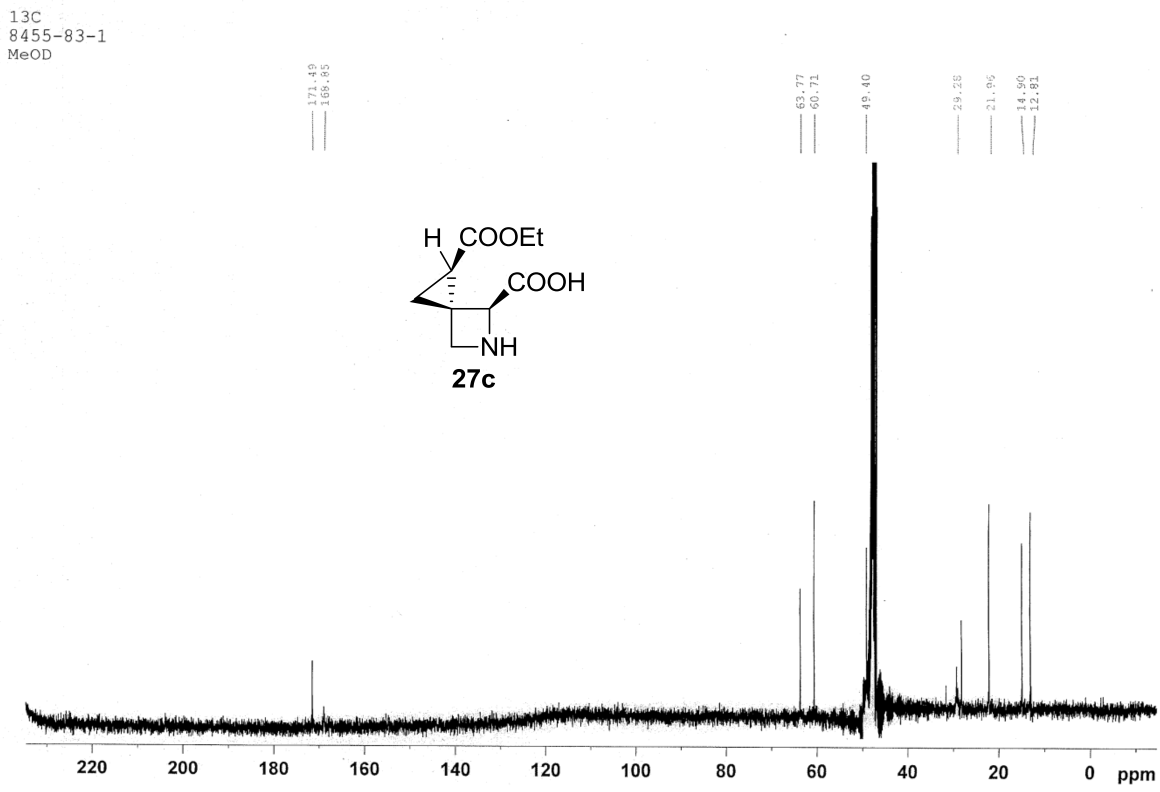
13C
8455-83-1
MeOD
171.49
168.85
63.77
60.71
49.40
29.28
21.96
14.90
12.81
H COOEt
COOH
NH
27c
220
200
180
160
140
120
100
80
60
40
20
0
ppm
